# A new integrative learning framework for integrating multiple secondary outcomes into primary outcome analysis: a case study on liver health

**DOI:** 10.1093/jrsssb/qkaf081

**Published:** 2025-12-30

**Authors:** Daxuan Deng, Peisong Han, Shuo Chen, Ming Wang, Chixiang Chen

**Affiliations:** Department of Public Health Sciences, Penn State College of Medicine, Hershey, PA 17033, USA; Biostatistics Innovation Group, Gilead Sciences, Foster City, CA 94404, USA; Division of Biostatistics and Bioinformatics, Department of Epidemiology and Public Health, University of Maryland School of Medicine, Baltimore, MD 21201, USA; Department of Population and Quantitative Health Sciences, Case Western Reserve University, Cleveland, OH 44106, USA; Division of Biostatistics and Bioinformatics, Department of Epidemiology and Public Health, University of Maryland School of Medicine, Baltimore, MD 21201, USA

**Keywords:** data integration, principal components, statistical learning

## Abstract

In the era of big data, secondary outcomes have become increasingly important alongside primary outcomes. These secondary outcomes, which can be derived from traditional endpoints in clinical trials, compound measures, or risk prediction scores, hold the potential to enhance the analysis of primary outcomes. Our method is motivated by the challenge of utilizing multiple secondary outcomes, such as blood biochemistry markers and urine assays, to improve the analysis of the primary outcome related to liver health. Current integration methods often fall short, as they impose strong model assumptions or require prior knowledge to construct over-identified working functions. This article addresses these challenges and opens a new avenue in data integration by introducing a novel integrative learning framework applicable in a general setting. The proposed framework allows for the robust, data-driven integration of information from multiple secondary outcomes, promotes the development of efficient learning algorithms, and ensures optimal use of available data. Extensive simulation studies demonstrate that the proposed method significantly reduces variance in primary outcome analysis, outperforming existing integration approaches. Additionally, applying this method to UK Biobank reveals that cigarette smoking is associated with increased fatty liver measures, with these effects being particularly pronounced in the older adult cohort.

## 1 Introduction

In traditional biomedical research, the primary outcome, also referred to as the primary endpoint in clinical studies, is the most important variable measured to assess whether a treatment or exposure being tested has had a significant effect. Secondary outcomes, on the other hand, are additional variables measured in the same study that provide overlapping or supplementary information about the treatment or exposure, including safety and tolerability, qualify of life, relevant biomarkers, and broader impacts on health and well-being (Levit et al., 2024). Compared to the primary outcome, secondary outcomes have historically received less attention but have become increasingly seen in contemporary biomedical studies. Here, we summarize three sources where secondary outcomes can be defined:

Type 1—Clinical endpoints. For example, in a clinical trial or epidemiological study evaluating a new medication for hypertension (Egan et al., 2016), the primary outcome could be the reduction in systolic blood pressure after 12 weeks of treatment. Secondary outcomes could include changes in diastolic blood pressure, changes in cholesterol levels, adherence to medication, etc. This is the most classic source of secondary outcomes in the literature. The following two types are less conventional but are increasingly observed in data science research using real-world or omics data.

Type 2—Compound indices defined by experts or leveraging over multi-domain diseases. For example, in studies of older adults, individuals may be classified as frail if they meet three or more of the following criteria: unintentional weight loss, exhaustion, weakness, slow walking speed, and low physical activity (Bandeen-Roche et al., 2015). As a result, in addition to frailty as the primary outcome to study the vulnerability of older adults, the presence of each individual component can be regarded as a secondary outcome.

Type 3—Data-driven risk scores. For example, the neurobiological risk score is a composite measure that quantifies an individual’s risk of developing a neurological or psychiatric disorder based on a combination of T1-weighted and diffusion tensor imaging measures (Rodrigue et al., 2023). This score can be constructed by weighting each factor based on its relative importance and contribution to the overall risk, with weights estimated using data science tools, including XGBoost (Ke et al., 2017). Hence, all individual factors used to construct risk scores (the primary outcome) can serve as secondary outcomes.

Despite being termed ‘secondary’, these outcomes have the potential to enhance estimation and inference in primary outcome analysis. This is particularly true for the latter two sources, where the primary outcome is a summary score derived from secondary outcomes and thus lacks the granular information captured by each secondary outcome. Relying solely on the primary outcome may not fully exploit the information of biological disease mechanisms or specific phenotypes from individual secondary outcomes. Therefore, there is a strong need to study methods that integrate information from secondary outcomes into the primary outcome analysis.

The case study in this article focuses on two primary outcomes: liver proton density fat fraction (PDFF), a quantitative imaging biomarker that measures hepatic fat content (Xia et al., 2023), and a new liver-biological risk score (LBRS), derived by modelling liver PDFF using predictive factors, such as blood measurements and urine assays. The latter outcome serves as a surrogate marker for liver PDFF and is particularly useful in scenarios where high-quality image markers require substantial effort and may provide an ‘easy pass’ for the timely identification of high-risk subjects and assessment of lifestyle behaviours influencing liver health (see Section 2.1 for more discussion). By studying liver PDFF/LBRS as primary outcomes, respectively, we aim to study the impact of cigarette smoking and explore whether this effect varies across different age groups. While recent studies have linked smoking to chronic liver disease (Marti-Aguado et al., 2022), its role in fatty liver remains poorly understood. Given the serious health consequences associated with fatty liver disease (Xia et al., 2023), there is a pressing need to investigate this relationship and provide evidence-based guidance for prevention. Beyond clinical relevance, we also pose a methodological question: can secondary outcomes, such as blood biochemistry factors and urine assays, contribute meaningfully to the analysis of the primary outcome? A plausible answer is yes, considering that these secondary outcomes are naturally associated with the primary outcome, even after adjusting for other covariates. However, a rigorous statistical tool to harness this association and robustly integrate information from multiple secondary outcomes remain under-explored.

From a statistical methodology perspective, the main goal of integrating information from secondary outcomes is to improve estimation efficiency and increase statistical power, without compromising estimation consistency, in the analysis of the primary outcome. Achieving this goal poses a significant challenge: how to leverage the association between primary and secondary outcomes without relying on strong assumptions, such as assuming shared effect sizes across outcomes (C. Chen, Wang, et al., 2023). One possible solution is to specify a joint likelihood for both primary and secondary outcomes by jointly modelling their means and associations (Alsefri et al., 2020; Ezzalfani et al., 2019). However, this approach is often computationally intensive, especially when outcomes follow distinct distributions. More importantly, it does not differentiate the roles of primary and secondary outcomes, which may yield bias in the primary analysis if the joint model is mis-specified. Therefore, it is desirable to develop methods that preserve robust and unbiased inference of the primary outcome while integrating information from secondary outcomes to improve estimation efficiency in the primary analysis, without treating all outcomes as equally important. In this context, C. Chen et al. (2022) proposed a re-weighting estimation scheme called the Vertical Information Scores (VIS) estimator to borrow information from a secondary outcome in a robust and computationally efficient manner. Here, ‘robust’ means that the mis-specification of the secondary outcome model will not introduce estimation bias to the primary outcome analysis. Further, C. Chen, Wang, et al. (2023) extended the work to accommodate multiple secondary outcomes, with other variants addressing missing data (Deng et al., 2024) and analysing survival outcomes (C. Chen, Yu, et al., 2023). Despite these strengths, these methods require a user-specified, over-identified estimating function, where the number of estimating function is larger than the number of parameter (Owen, 2001). While over-identification could be achieved in repeatedly measures settings by stacking multiple estimating functions with varying base matrices (C. Chen et al., 2022), it remains unclear for a general data setting, limiting broader applicability. Before describing our solutions, we note that other advanced methods have been developed for integrating external information into internal analysis (Chatterjee et al., 2016; C. Chen et al., 2024; Duan et al., 2022; Han et al., 2024; Zhai & Han, 2024; H. Zhang et al., 2020). However, these methods are designed for combining independent studies and are not applicable to our setting, where both primary and secondary outcomes are drawn from the same study.

**Figure 1.**
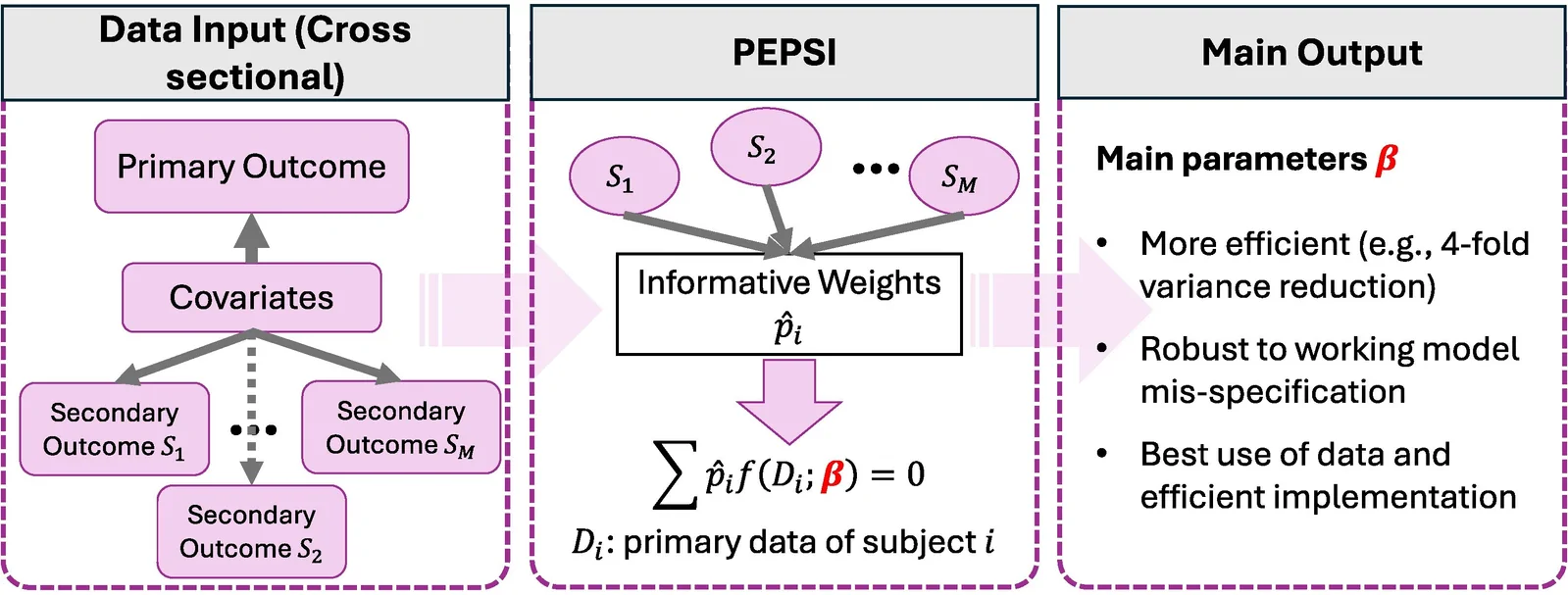
Overall workflow of PEPSI. Dashed line in the left panel implies that covariates have partial or no effect on the secondary outcome.

In this article, we propose a new integrative learning framework named PEPSI, *P*enalized *E*mpirical likelihood and *P*rincipal component analysis-based *S*econdary outcome *I*ntegrative learning. PEPSI is designed primarily (but not limited to) for settings with multiple cross-sectional secondary outcomes, and it innovatively integrates data science techniques, including constrained optimization, penalized empirical likelihood, and principal component analysis, to achieve three goals (Figure 1). (1) PEPSI robustly integrates information from secondary outcomes in a data-driven manner, without the need for prior knowledge to specify over-identified functions, a major limitation of existing methods (C. Chen, Wang, et al., 2023; Wolf et al., 2024). The key to achieving this is learning zero coefficients in secondary outcome models. Notably, efficiency gains occur when some coefficients in certain secondary models are zero, but users do not need to know which coefficients from which secondary outcome models are zeros. Even when all covariate effects are non-zero, PEPSI performs at least as well as methods without data integration. As a result, PEPSI preserves the original estimand or the hypothesis of interest defined by the primary outcome analysis or study protocol. (2) PEPSI efficiently accommodates multiple secondary outcomes through a computationally efficient learning algorithm. (3) Within certain classes of estimators, PEPSI optimally integrate multiple secondary outcomes, demonstrating superior performance over existing integration schemes (C. Chen, Wang, et al., 2023). Substantial simulation studies also demonstrate PEPSI’s advantages, showing substantial reductions in estimation variability with negligible estimation bias. Moreover, the method significantly enhances the analysis studying the impact of cigarette smoking on liver health. Notably, compared with the classic estimator, PEPSI achieves over fourfold relative efficiency in many estimates and identifies several significant factors influencing the LBRS outcome, such as age ≥65 and its interaction with smoking, suggesting heterogeneous smoking effects.

## 2 Preliminaries

### 2.1 Liver-biological risk scores

Recent literature revealed that PDFF was linked to significantly elevated risks for liver cancer, non-alcoholic steatohepatitis, cirrhosis, and other forms of liver disease (Xia et al., 2023). However, obtaining accurate liver PDFF, captured by MRI-based water-fat separation technique (Starekova et al., 2021), can be time-consuming, expensive, and is known to have high sampling variability (Kramer et al., 2017). On the other hand, blood biochemistry measures and urine biomarkers are much easier and cheaper to obtain and have been shown to be associated with chronic liver conditions (Kwo et al., 2017; Longo et al, 2012). Therefore, as one of the application goals in this article, we are interested in constructing LBRS and studying their utility compared to the liver PDFF measure. As illustrated in Section 5, we constructed the LBRS by predicting PDFF from multiple blood and urine biomarkers using a machine learning model. The constructed LBRS can also be interpreted as a de-noised outcome by projecting liver PDFF onto the space of informative biomarkers. This risk score may capture the underlying information of liver PDFF explained by the biomarkers and thus has the potential to provide an ‘easy pass’ and possibly strong signals for the timely identification of high-risk subjects, assessment of environmental factors and lifestyle behaviours influencing LBRS, and examination of their heterogeneous effects among subcohorts without requiring the availability of liver PDFF measures. The results will also inform future trial designs to infer definitive conclusions.

After constructing LBRS, our clinical interest is to study and compare the impact of cigarette smoking on LBRS and liver PDFF, and to explore whether this effect differs across age groups, a topic that remains understudied in the literature. Therefore, our primary outcomes are LBRS and liver PDFF, with cigarette smoking as the primary exposure. Our secondary outcomes include 22 blood biochemistry measures and 3 urine assays, which were selected by domain experts and are expected to be strongly associated with the two primary outcomes. Given the presence of multiple secondary outcomes, our methodological objective is to develop an effective integrative learning strategy that enhances estimation efficiency in the primary analysis by robustly leveraging information from these secondary outcomes.

### 2.2 Notations and basic set-up

We now formally introduce the primary and secondary data in a generic setting, of which our data application is one special scenario. Specifically, let $D_{i}=\{Y_{i},X_{i}\}$, i=1,2,…,n, be independent and identically distributed (i.i.d.) primary data for the primary analysis, where *Y* and X are the primary outcome and covariate vector, respectively, and *n* is the sample size. We denote $\hat{\beta }$ as the estimated parameter vector, which can be obtained by solving the estimating equation $\sum_{i=1}^{n}f(D_{i};\beta )=0$. The function f(⋅) can be any regular estimating function, such as the first derivative of a least squares loss function, a score function, or a function from generalized estimating equations (Van der Vaart, 2000). The limiting value of $\hat{\beta }$, denoted by $\beta_{0}$, satisfies $E\{f(D;\beta_{0})\}=0$. In Section 2.1, β contains the effects of risk factors, such as cigarette smoking, age, and their interaction term, on LBRS/PDFF.

On the other hand, we may have M≥1 i.i.d. secondary data ${\tilde{D}}_{mi}=\{{\tilde{Y}}_{mi},{\tilde{X}}_{mi}\}$, for i=1,2,…,n and m=1,…,M, collected from the same cohort of participants, where ${\tilde{Y}}_{m}$ and ${\tilde{X}}_{m}$ denote the secondary outcome and covariate vector in the *m*th secondary data, respectively. We emphasize that the primary and secondary data are not independent of each other. In our case study, for instance, ${\tilde{Y}}_{m}$ includes blood and urine biomarkers that are believed to be associated with and predictive of liver health (e.g. LBRS/PDFF). Throughout this article we focus on the setting where both primary and secondary data are cross-sectional and share the same covariates, i.e. $X={\tilde{X}}_{m}$, for all m=1,…,M, which aligns with our application. However, the proposed method can be extended to more general settings.

Before introducing the method, we briefly explain how the variables described above are typically determined in practice. The primary outcome and the corresponding hypothesis should be defined prior to the analysis stage, while secondary outcomes are typically pre-specified by the study protocol or defined from the construction of the primary outcome. In contrast, covariates should be selected independent of secondary outcomes, based on the original research objectives related to the primary outcome. In our case study, the primary outcome was constructed using a set of informative biomarkers selected by clinical collaborators. Consequently, the secondary outcomes naturally consisted of these variables. This selection process took place during the design phase and was informed by domain expertise. We refer readers to Section 3.2 for general guidelines on selecting secondary outcomes. Covariates, on the other hand, were chosen according to the specific research question. For instance, if examining sex differences in liver PDFF is of interest, sex would be included as a covariate and excluded from the pool of secondary outcomes.

In the following section, we describe a novel integrative learning approach that incorporates information from all secondary data into the primary data analysis in a data-driven and robust manner, without altering the original estimand or the hypothesis related to $\beta_{0}$ defined by the primary outcome analysis or study protocol. To smoothly introduce our method, we start with the setting where there is only one secondary data and then illustrate the setting of multiple secondary data.

## 3 The PEPSI framework

### 3.1 Single secondary data

We first outline the learning procedure and then explain the underlying rationale behind the method. Suppose there is only one secondary data available in the study, denoted by ${\tilde{D}}_{m}$, for some *m* satisfying 1≤m≤M. To enhance parameter estimation and inference by integrating information from the secondary data, we propose a new estimator, named the Penalized empirical likelihood-based Secondary outcome Integrative learning (PSI) estimator, a special case of PEPSI, denoted by ${\hat{\beta }}_{\mathrm{PSI}}$, which is obtained by solving the following re-weighted estimating equation:


(1) $$\sum_{i=1}^{n}{\hat{\;p}}_{mi}f(D_{i};\beta )=0.$$

The weights are proposed to be ${\hat{\;p}}_{mi}=n^{-1}\{1+\lambda_{m}^{T}({\hat{\theta }}_{m})g_{m}({\tilde{D}}_{mi};{\hat{\theta }}_{m}){\}}^{-1}$, for i=1,…,n, where $\lambda_{m}(\theta_{m})$ is a function of $\theta_{m}$ satisfying $n^{-1}\sum_{i=1}^{n}\{1+\lambda_{m}^{T}g_{m}({\tilde{D}}_{mi};\theta_{m}){\}}^{-1}g_{m}({\tilde{D}}_{mi};\theta_{m})=0$. The function $g_{m}({\tilde{D}}_{m};\theta_{m})$ is a ‘just/over-identified’ working estimating function for the secondary data, parameterized by $\theta_{m}$, which satisfies $E\{g_{m}({\tilde{D}}_{m};\theta_{m}^{*})\}=0$ for some value $\theta_{m}^{*}$. Let the dimensions of $g_{m}({\tilde{D}}_{m};\theta_{m})$ and $\theta_{m}$ be $r_{m}$ and $p_{m}$, respectively. Here, ‘just/over-identified’ means that the length of the estimating function vector is equal to or greater than the length of the parameter vector, i.e. $r_{m}\geq p_{m}$ (Owen, 2001). In practice, an over-identified estimating function within a regression framework can be constructed by setting certain components of ${\hat{\theta }}_{m}$ to exactly zero when it is known that the corresponding covariates have no effect on the secondary outcome. However, we highlight here that prior knowledge of constructing the over-identified function is not required to implement the PSI framework.

Moreover, ${\hat{\theta }}_{m}$ is obtained by minimizing the following penalized loss function (Leng & Tang, 2012):


(2) $$l_{\;pm}(\theta_{m})=\sum_{i=1}^{n}\log\;\{1+\lambda_{m}^{T}(\theta_{m})g_{m}({\tilde{D}}_{mi};\theta_{m})\}+n\sum_{\;j=1}^{\;p_{m}}\rho_{\tau_{m}}(|\theta_{mj}|).$$

Here, the first term $l_{m}(\theta_{m})=\sum_{i=1}^{n}\log\;\{1+\lambda_{m}^{T}g_{m}({\tilde{D}}_{mi};\theta_{m})\}$ corresponds to the negative logarithm of the empirical likelihood ratio function (Qin & Lawless, 1994), while the second term involves a penalty function $\rho_{\tau_{m}}(\cdot )$ with a tuning parameter $\tau_{m}$, where $\{\theta_{mj}{\}}_{\;j=1}^{\;p_{m}}$ are the entries of the vector $\theta_{m}$. We sometimes write ${\hat{\theta }}_{m}={\hat{\theta }}_{m,\tau_{m}}$ and $l_{\;pm}(\theta_{m})=l_{\;pm}(\theta_{m,\tau_{m}})$ to emphasize the dependency on $\tau_{m}$. In this article, we use the SCAD penalty (Fan & Li, 2001) for illustration, though other penalty functions can be applied (C.-H. Zhang, 2010; Zou, 2006). Therefore, the proposed weights ${\hat{\;p}}_{mi}$ can be interpreted as an extension of the empirical likelihood framework (Qin & Lawless, 1994), where the parameters are estimated using a penalized procedure.

We now provide an intuitive rationale for how the proposed PSI estimator works: The constructed weights $\{{\hat{\;p}}_{mi}{\}}_{i=1}^{n}$ summarize useful information from the secondary data and serve as a bridge connecting the primary and secondary data, while the proposed penalized optimization aims to identify zero-valued parameters in $\theta_{m}$. When the function $g_{m}({\tilde{D}}_{mi};\theta_{m})$ is originally just identified and the penalized regression does not identify any zero-valued parameters in $\theta_{m}$, the weights reduce to 1/n, i.e. non-informative weights (C. Chen et al., 2022; Qin & Lawless, 1994). Under this scenario, there is no efficiency gain for estimating β. However, if any zero components of $\theta_{m}$ are identified, the efficiency gain can be substantial. Intuitively, shrinkage of very small estimated parameter values to zero implicitly facilitates the construction of over-identified working functions in $g_{m}({\tilde{D}}_{mi};\theta_{m})$, which in turn promotes a more efficient estimate of the empirical distribution (C. Chen et al., 2022; Qin & Lawless, 1994). As a result, the weights in (1) incorporate additional information from the secondary data, thereby enhancing the estimation of β in the primary analysis. It is worth noting that the existence of null effects from certain covariates on the secondary outcome is common in real practice. In Section 5, for example, we observed that smoking had little effect on several secondary outcomes, such as calcium in blood, potassium in urine. Consistently, existing literature provide limited evidence supporting significant effects of smoking on these outcomes after adjusting for other covariates (Breitling, 2015). Importantly, regardless of covariate effects in secondary outcome models, our method does not impose any assumptions on the true effect sizes from the primary outcome model, thus maintaining substantial flexibility in applications. In summary, the proposed PSI facilitates data integration by auto-constructing over-identified working functions through the data-driven identification of zero components in $\theta_{m}$. For further details, see Theorem 1 and the accompanying discussion.

The calculation of the weights $\{{\hat{\;p}}_{mi}{\}}_{i=1}^{n}$ involves constrained optimization combined with a penalty, which could be computationally intensive. To promote an efficient computation, we adopt a quadratic approximation for the penalty function in (2) and iteratively update $\theta_{m}$ and $\lambda_{m}$ iteratively. During optimization, we set entries of $\theta_{m}$ to zero if their magnitudes fall below a pre-specified threshold *γ*, which is set to $1\times 10^{-3}$ in both simulations and application. The tuning parameter $\tau_{m}$ is selected by minimizing the following Bayesian information criteria: $\text{BIC}(\tau_{m})=2l_{m}({\hat{\theta }}_{m,\tau_{m}})+C_{m}\log\;(n)\sum_{\;j=1}^{\;p_{m}}I({\hat{\theta }}_{mj,\tau_{m}}\neq 0)$, where ${\hat{\theta }}_{m,\tau_{m}}$ are obtained by minimizing (2) under $\tau_{m}$, and $C_{m}$ is a hyper-parameter controlling the bias-variance trade-off in practice. As suggested by Leng and Tang (2012), setting $C_{m}=\max(\log\;\log\;p_{m},1)$ provides a reasonable choice. The pseudo algorithm for implementing PSI is summarized in Algorithms 1 and 2.

**Algorithm 1:** Algorithm for obtaining the penalized empirical likelihood estimator

Input: secondary data $\{{\tilde{D}}_{mi}{\}}_{i=1}^{n}.$
**for all** $\tau_{m}$ **do**
${\hat{\lambda }}_{m}\leftarrow 0.$
**repeat**
${\hat{\theta }}_{m,\tau_{m}}\leftarrow \arg\;\min_{\theta_{m,\tau_{m}}}l_{\;pm}(\theta_{m,\tau_{m}}).$
Update ${\hat{\lambda }}_{m}$ by solving $\sum_{i=1}^{n}\{1+{\hat{\lambda }}_{m}^{T}g_{m}({\tilde{D}}_{mi};{\hat{\theta }}_{m,\tau_{m}}){\}}^{-1}g_{m}({\tilde{D}}_{mi};{\hat{\theta }}_{m,\tau_{m}})=0.$
**for all** *j* **do**
${\hat{\theta }}_{mj,\tau_{m}}\leftarrow {\hat{\theta }}_{mj,\tau_{m}}I(|{\hat{\theta }}_{mj,\tau_{m}}|\leq \gamma ).$
**end for**
**until** ${\hat{\theta }}_{m,\tau_{m}}$ converges.
**end for**
${\hat{\tau }}_{m}\leftarrow \arg\;\min_{\tau_{m}}\text{BIC}(\tau_{m}).$
${\hat{\theta }}_{m}\leftarrow {\hat{\theta }}_{m,{\hat{\tau }}_{m}}.$
$\text{Output}:\;{\hat{\theta }}_{m}.$

**Algorithm 2:** Algorithm for obtaining the PSI estimator

Input: primary data $\{D_{i}{\}}_{i=1}^{n}$, secondary data $\{{\tilde{D}}_{mi}{\}}_{i=1}^{n}.$
Obtain ${\hat{\theta }}_{m}$ using Algorithm 1.
Obtain ${\hat{\lambda }}_{m}$ by solving $\sum_{i=1}^{n}\{1+{\hat{\lambda }}_{m}^{T}g_{m}({\tilde{D}}_{mi};{\hat{\theta }}_{m}){\}}^{-1}g_{m}({\tilde{D}}_{mi};{\hat{\theta }}_{m})=0$.
${\hat{\;p}}_{mi}\leftarrow n^{-1}\{1+{\hat{\lambda }}_{m}^{T}({\hat{\theta }}_{m})g_{m}({\tilde{D}}_{mi};{\hat{\theta }}_{m}){\}}^{-1}.$
Obtain ${\hat{\beta }}_{\mathrm{PSI}}$ by solving $\sum_{i=1}^{n}{\hat{\;p}}_{mi}f(D_{i};\beta_{\mathrm{PSI}})=0$.
$\text{Output}:\;{\hat{\beta }}_{\mathrm{PSI}}.$

To explore the validity of PSI, we study the asymptotic property of ${\hat{\beta }}_{\mathrm{PSI}}$ in Theorem 1. Without loss of generality, we write $\theta_{m}^{*}=(\theta_{m(1)}^{*T},\theta_{m(2)}^{*T}{)}^{T}$, where $\theta_{m(1)}^{*}$ and $\theta_{m(2)}^{*}$ are respective zero and non-zero components of $\theta_{m}^{*}$. Let the dimension of $\theta_{m(1)}^{*}$ be $q_{m}$, and $H_{m}$ be a matrix such that $H_{m}\theta_{m}^{*}=\theta_{m(1)}^{*}$.

Theorem 1 Under regularity conditions listed in the online supplementary material, we have ${\hat{\theta }}_{m(1)}=0$ with probability tending to one; ${\hat{\beta }}_{\mathrm{PSI}}$ is consistent to $\beta_{0}$; $\sqrt{n}({\hat{\beta }}_{\mathrm{PSI}}-\beta_{0})$ converges in distribution to normal distribution $N(0,V_{\mathrm{PSI}})$. Here $V_{\mathrm{PSI}}=\Gamma^{-1}(\Sigma -\Lambda_{m}S_{m}\Lambda_{m}^{T}-\Lambda_{m}P_{m}\Lambda_{m}^{T})\Gamma^{-1,T}$, where $\Gamma =E\{\partial f(D;\beta_{0})/\partial \beta^{T}\}$, $\Sigma =E\{f(D;\beta_{0})f^{T}(D;\beta_{0})\}$, $\Lambda_{m}=E\{f(D;\beta_{0})g_{m}^{T}({\tilde{D}}_{m};\theta_{m}^{*})\}$, $S_{m}=A_{m}^{-1}-A_{m}^{-1}$  $B_{m}C_{m}B_{m}^{T}A_{m}^{-1}$, $P_{m}=A_{m}^{-1}B_{m}C_{m}H_{m}^{T}(H_{m}C_{m}H_{m}^{T}{)}^{-1}H_{m}C_{m}B_{m}^{T}A_{m}^{-1}$, where $A_{m}=E\{g_{m}({\tilde{D}}_{m};\theta_{m}^{*})g_{m}^{T}({\tilde{D}}_{m};\theta_{m}^{*})\}$, $B_{m}=E\{\partial g_{m}({\tilde{D}}_{m};\theta_{m}^{*})/\partial \theta_{m}^{T}\}$, $C_{m}=(B_{m}^{T}A_{m}^{-1}$  $B_{m}{)}^{-1}$.

There are several implications regarding the efficiency of ${\hat{\beta }}_{\mathrm{PSI}}$. First, we compare the efficiency of ${\hat{\beta }}_{\mathrm{PSI}}$, ${\hat{\beta }}_{\mathrm{VIS}}$, and ${\hat{\beta }}_{\mathrm{naive}}$, where ${\hat{\beta }}_{\mathrm{VIS}}$ is the VIS estimator proposed by C. Chen et al. (2022) without adapting the penalized procedure, and ${\hat{\beta }}_{\mathrm{naive}}$ is the estimator that does not borrow information from the secondary data, obtained by solving $\sum_{i=1}^{n}f(D_{i};\beta )=0$. Note that $\Gamma^{-1}(\Sigma -\Lambda_{m}S_{m}\Lambda_{m}^{T})\Gamma^{-1,T}$ and $\Gamma^{-1}\Sigma \Gamma^{-1,T}$ are the asymptotic variances of ${\hat{\beta }}_{\mathrm{VIS}}$ and ${\hat{\beta }}_{\mathrm{naive}}$, respectively, with two matrices $S_{m}$ and $P_{m}$ being non-negative definite. The efficiency comparison among the three estimators is summarized below: in general, ${\hat{\beta }}_{\mathrm{PSI}}$ is more efficient than ${\hat{\beta }}_{\mathrm{VIS}}$, and ${\hat{\beta }}_{\mathrm{VIS}}$ is more efficient than ${\hat{\beta }}_{\mathrm{naive}}$. The superiority of ${\hat{\beta }}_{\mathrm{PSI}}$ over ${\hat{\beta }}_{\mathrm{naive}}$ arises from two aspects of variance reduction. The first comes from the term $\Gamma^{-1}\Lambda_{m}S_{m}\Lambda_{m}^{T}\Gamma^{-1,T}$, which is non-zero and non-negative definite if the estimating function $g_{m}({\tilde{D}}_{m};\theta_{m})$ is over-identified in its original construction. This can be achieved, for example, by forcing some components of ${\hat{\theta }}_{m}$ to be exactly zero when it is known that the corresponding covariates have no effect on the secondary outcome. This variance reduction term is also shared by ${\hat{\beta }}_{\mathrm{VIS}}$. However, it vanishes when no prior knowledge is available to construct an over-identified function. The second comes from the term $\Gamma^{-1}\Lambda_{m}P_{m}\Lambda_{m}^{T}\Gamma^{-1,T}$, which is achieved by identifying zero entries in $\theta_{m}^{*}$ in a data-driven manner, without requiring prior knowledge. This additional variance reduction explains the superiority of ${\hat{\beta }}_{\mathrm{PSI}}$ over ${\hat{\beta }}_{\mathrm{VIS}}$.

The efficiency improvement of ${\hat{\beta }}_{\mathrm{PSI}}$ also depends on the association between the primary and secondary data, measured by $\Lambda_{m}$. A stronger association leads to greater variance reduction, whereas if the data are independent, $\Lambda_{m}$ becomes zero and the secondary data do not contribute to the primary data analysis. Note that $\Lambda_{m}$ depends not only on the association between *Y* and ${\tilde{Y}}_{m}$ but also on the relationship between X and ${\tilde{X}}_{m}$. For example, when both the primary and secondary outcomes are cross-sectional and follow generalized linear models, $\Lambda_{m}=E\{\text{cov}(Y,{\tilde{Y}}_{m}\;|\;X,{\tilde{X}}_{m})X{\tilde{X}}_{m}^{T}\}$. Therefore, it is recommended to always include X as part of ${\tilde{X}}_{m}$, as this generally enhances integration performance.

Moreover, the efficiency superiority of ${\hat{\beta }}_{\mathrm{PSI}}$ is robust to misspecification of the secondary working model. Since inference on the secondary data is not of direct interest, $g_{m}({\tilde{D}}_{m};\theta_{m})$ does not need to be correctly specified, nor does $\theta_{m}^{*}$ need to represent the true parameter generating the secondary data. Instead, it is sufficient that there exists some $\theta_{m}^{*}$ satisfying $E\{g_{m}({\tilde{D}}_{m};\theta_{m}^{*})\}=0$. This robustness property is demonstrated through numerical experiments in Section 4.1.

Finally, if all coefficients in $\theta_{m}^{*}$ are non-zero, which may happen with a single secondary data with a limited set of covariates, and no prior knowledge is available to guide the construction of the over-identified function, the PSI estimator will not yield efficiency gains. However, this limitation can be mitigated by using multiple secondary outcomes, where some coefficients in $\theta_{m}^{*}$ may be zero for certain *m*, but not for all *m*. This naturally motivates the need for a new method to integrating multiple secondary outcomes, as described in the following section.

### 3.2 Multiple secondary data

The PSI framework provides the essential foundation for the PEPSI framework, which addresses a key research challenge: how to effectively leverage multiple secondary data sources to enhance statistical performance in the primary analysis. In our data application, we have 22 blood biochemistry markers and 3 urine biomarkers in addition to the primary outcome, all of which are highly associated with liver health. Integrating information from these multiple secondary outcomes can significantly improve the primary outcome analysis.

Suppose there are *M* secondary data $\{{\tilde{D}}_{m}{\}}_{m=1}^{M}$, and we aim to integrate all of them into the primary data analysis. A natural approach is to stack all working estimating functions $\{g_{m}({\tilde{D}}_{m},\theta_{m}){\}}_{m=1}^{M}$ and form a pooled estimating function $G(\tilde{D};\Theta )=(g_{1}^{T}({\tilde{D}}_{1};\theta_{1}),\ldots ,g_{M}^{T}({\tilde{D}}_{M};\theta_{M}){)}^{T},$ where $\tilde{D}=\{{\tilde{D}}_{1},\ldots ,{\tilde{D}}_{M}\}$ and $\Theta =(\theta_{1}^{T},\ldots ,\theta_{M}^{T}{)}^{T}$ are the pooled data and pooled parameter vector, respectively. One could then construct a PSI estimator, as described in Section 3.1, with $g_{m}({\tilde{D}}_{m},\theta_{m})$ replaced by $G(\tilde{D};\Theta )$. However, this approach becomes computationally challenging due to the high dimensions of both $G(\tilde{D};\Theta )$ and Θ, as well as the strong association and potential co-linearity among functions in $G(\tilde{D};\Theta )$. These features hinder the broad applicability of joint estimation. For example, suppose we have 25 secondary outcomes that may exhibit substantial correlation. Even if each secondary outcome model contains only 4 parameters, this still yields 100 functions in the $G(\tilde{D};\Theta )$ vector and 100 parameters in Θ. The dimensionality grows much larger when the number of secondary outcomes or parameters in each model increases. Rather than solving all parameters in Θ simultaneously, we adopt a divide-and-conquer strategy. Specifically, we obtain estimators $\{{\hat{\theta }}_{m}{\}}_{m=1}^{M}$ from each secondary data separately, as described in Section 3.1, and then pool them to form the enlarged estimator vector $\hat{\Theta }$. This alternative avoids the computationally intensive task of solving the entire parameter space within a single penalized and constrained optimization step, thereby substantially reducing the computational load.

Despite the computational relief gained from estimating Θ, the dimension of the enlarged vector $G(\tilde{D};\hat{\Theta })$ remains high. More importantly, because the plug-in estimator $\hat{\Theta }$ is obtained via the divide-and-conquer approach rather than by directly solving the pooled estimating equation, it becomes unclear how the resulting $G(\tilde{D};\hat{\Theta })$ would contribute to the primary data analysis. Consequently, the weights defined in Section 3.1 may not be directly applicable.

One strategy for integrating multiple secondary outcomes is to design an Averaging scheme that takes a linear combination of $\{{\hat{\;p}}_{mi}{\}}_{m=1}^{M}$, obtained by PSI across the secondary outcomes. Specifically, the Averaging scheme estimator ${\hat{\beta }}_{\mathrm{avg}}$ is defined as the solution to the estimating equation $\sum_{i=1}^{n}(\sum_{m=1}^{M}w_{m}{\hat{\;p}}_{mi})f(D_{i};\beta )=0$, where the hyperparameters $\{w_{m}{\}}_{m=1}^{M}$, satisfying $\sum_{m=1}^{M}w_{m}=1$, are the weights assigned for each secondary outcome and can be determined via data-driven optimization (C. Chen, Wang, et al., 2023). Details of this method are described in Section 1 in the online supplementary material. We note that ${\hat{\beta }}_{\mathrm{avg}}$ is new in that it combines the strengths of the proposed PSI and the MinBo approach (C. Chen, Wang, et al., 2023). However, a potential limitation is that it may not optimally exploit the information from the secondary outcomes. In what follows, we introduce another new perspective, PEPSI, which constructs weights by adapting dimensionality reduction and projection techniques to address this limitation.

To motivate the construction of new weights, we examine the asymptotic formula $\text{var}\{n^{-1/2}\sum_{i=1}^{n}g_{m}({\tilde{D}}_{mi};{\hat{\theta }}_{m})\}=R_{m}A_{m}R_{m}^{T}+o_{p}(1),$ where $R_{m}=A_{m}(S_{m}+P_{m})$ with $A_{m}$, $S_{m}$, and $P_{m}$ defined in Theorem 1. We notice here that the rank of $R_{m}$ is $r_{m}-(p_{m}-q_{m})$, so is the rank of $R_{m}A_{m}R_{m}^{T}$, where $r_{m}$, $p_{m}$, and $q_{m}$ are the dimensions of $g_{m}({\tilde{D}}_{m};\theta_{m})$, $\theta_{m}$ and $\theta_{m(1)}^{*}$ (defined in Section 3.1), respectively. This rank can be understood in terms of two components: $r_{m}$ and $p_{m}-q_{m}$. The first component, $r_{m}$, reflects the total degrees of freedom (DoF) in $g_{m}({\tilde{D}}_{m};\theta_{m}^{*})$ when the parameters in $\theta_{m}^{*}$ are fully known. The second component, $\left(p_{m}-q_{m}\right)$, represents the loss of DoF due to estimating the non-zero components of $\theta_{m}^{*}$. Thus, the difference between these two components indicates the remaining DoF that can contribute to the primary analysis. In other words, there exists a low-dimensional representation within $G(\tilde{D};\hat{\Theta })$, referred to as the ‘informative component’, which plays an essential role in data integration. This observation motivates us to locate the informative component that benefits primary model inference by reducing the dimensionality of $G(\tilde{D};\hat{\Theta })$. To this end, we propose an extended PSI framework, named PEPSI, which involves two steps for weight construction.

**Step 1 (dimension reduction)**: We apply principal component analysis (PCA) to $\hat{R}G({\tilde{D}}_{i};\hat{\Theta })$, i=1,2,…,n, where $R=\text{diag}(R_{1},\ldots ,R_{M})$, and $\hat{R}$ is the sample estimate of R. Let $\hat{K}$ be the rank of the sample variance matrix, approximated by $\sum_{m=1}^{M}r_{m}-(p_{m}-{\hat{q}}_{m})$ based on the above discussion, where ${\hat{q}}_{m}$ is the number of zero entries in ${\hat{\theta }}_{m(1)}$. Let $\hat{\Phi }$ and $\hat{U}$ be the diagonal matrix of the first $\hat{K}$ eigenvalues and the corresponding rotation matrix of eigenvectors obtained from PCA.

**Step 2 (weight construction)**: We then construct the weights using the following expression to ensure valid data integration: ${\hat{\;p}}_{i}=n^{-1}\{1-n^{-1}G^{T}({\tilde{D}}_{i};\hat{\Theta }){\hat{R}}^{T}\hat{U}{\hat{\Phi }}^{-1}{\hat{U}}^{T}\hat{R}\sum_{i=1}^{n}G({\tilde{D}}_{i};\hat{\Theta })\}.$ The heuristic explanation behind constructing these weights is to project f(D;β) onto the orthogonal complement of linear space spanned by $G(\tilde{D};\hat{\Theta })$, thereby minimizing the estimator variance (see Corollary 1 and its proof in the online supplementary material for technical details). After weight construction, the PEPSI estimator ${\hat{\beta }}_{\mathrm{PEPSI}}$ is obtained by solving the re-weighted estimating equation: $\sum_{i=1}^{n}{\hat{\;p}}_{i}f(D_{i};\beta )=0$.

The pseudo algorithm for implementing PEPSI is provided in Algorithms 1 and 3, and the asymptotic properties of ${\hat{\beta }}_{\mathrm{PEPSI}}$ are summarized in Theorem 2.

**Algorithm 3:** Algorithm for obtaining the PEPSI estimator

Input: primary data $\{D_{i}{\}}_{i=1}^{n}$, secondary data $\{{\tilde{D}}_{i}{\}}_{i=1}^{n}.$
Obtain $\{{\hat{\theta }}_{m}{\}}_{m=1}^{M}$ using Algorithm 1 and set $\hat{\Theta }\leftarrow ({\hat{\theta }}_{1}^{T},\ldots ,{\hat{\theta }}_{M}^{T}{)}^{T}$.
Obtain $\hat{\Phi }$ and $\hat{U}$ by applying PCA on $\hat{R}G({\tilde{D}}_{i};\hat{\Theta })$.
${\hat{\;p}}_{i}\leftarrow n^{-1}\{1-n^{-1}G^{T}({\tilde{D}}_{i};\hat{\Theta }){\hat{R}}^{T}\hat{U}{\hat{\Phi }}^{-1}{\hat{U}}^{T}\hat{R}\sum_{i=1}^{n}G({\tilde{D}}_{i};\hat{\Theta })\}.$
Obtain ${\hat{\beta }}_{\mathrm{PEPSI}}$ by solving $\sum_{i=1}^{n}{\hat{\;p}}_{i}f(D_{i};\beta_{\mathrm{PEPSI}})=0$.
$\text{Output}:\;{\hat{\beta }}_{\mathrm{PEPSI}}.$

Theorem 2 Under mild conditions listed in the online supplementary material, ${\hat{\beta }}_{\mathrm{PEPSI}}$ is consistent to $\beta_{0}$, and $\sqrt{n}({\hat{\beta }}_{\mathrm{PEPSI}}-\beta_{0})$ converges in distribution to $N(0,V_{\mathrm{PEPSI}})$. Here, $V_{\mathrm{PEPSI}}=\Gamma^{-1}(\Sigma -\Lambda R^{T}U\Phi^{-1}U^{T}R\Lambda^{T})\Gamma^{-1,T}$, where Γ and Σ are defined in Theorem 1; $\Lambda =E\{f(D;\beta_{0})G^{T}(\tilde{D};\Theta^{*})\}$; Φ and U are the diagonal matrix and rotation matrix containing the first *K* eigenvalues and eigenvectors of $W=RE\{G(\tilde{D};\Theta^{*})G^{T}(\tilde{D};\Theta^{*})\}R^{T}$, respectively; $\Theta^{*}$ is the pooled true value vector satisfying $E\{G(\tilde{D};\Theta^{*})\}=0$ and *K* is the rank of W.

Theorem 2 shows that the PEPSI framework effectively reduces the variance in estimating β. A key insight is that identifying zero coefficients is essential for leveraging additional information, and incorporating multiple secondary outcomes, rather than relying on a single one, is beneficial, as it increases the likelihood of encountering zero coefficients across various secondary models. In other words, as long as some coefficients in $\theta_{m}^{*}$ are zero for at least one *m*, and more importantly, without requiring prior knowledge of which secondary outcomes contain zero coefficients, the PEPSI estimator is more likely to achieve improved efficiency.

Corollary 1 Given a collection of working estimating functions $\{g_{m}({\tilde{D}}_{m};\theta_{m}){\}}_{m=1}^{M}$ and penalized empirical likelihood estimates $\{{\hat{\theta }}_{m}{\}}_{m=1}^{M}$ from (2), ${\hat{\beta }}_{\mathrm{PEPSI}}$ is the most efficient estimator among the following class of estimating equations

$$\left\{\frac{1}{n}\sum_{i=1}^{n}\left\{f(D_{i};\beta )+\sum_{m=1}^{M}F_{m}g_{m}({\tilde{D}}_{mi};{\hat{\theta }}_{m})\right\}=o_{p}(n^{-1/2})\right\},$$

where $\{F_{m}{\}}_{m=1}^{M}$ are any arbitrary conformable matrices.

In addition to investigating the asymptotic distributions, we have examined other theoretical properties of the PSI/PEPSI estimators (see Section 1 of the online supplementary material). Corollary 1 shows the optimality of PEPSI estimator within a specific class of estimators, including ${\hat{\beta }}_{\mathrm{avg}}$. This analysis highlights the estimator’s ability to achieve the smallest variance and thus demonstrates that PEPSI effectively leverages secondary outcomes to maximize efficiency. It is also worth noting that ${\hat{\beta }}_{\mathrm{PSI}}$ and ${\hat{\beta }}_{\mathrm{PEPSI}}$ are equivalent when there is only a single secondary outcome. Hence, ${\hat{\beta }}_{\mathrm{PEPSI}}$ can be viewed as a generalization of ${\hat{\beta }}_{\mathrm{PSI}}$.

We conclude the method section with a discussion on secondary outcome integration within the PEPSI framework. Although the proposed framework incorporates both primary and secondary outcomes into the analysis, our theoretical results show that, under mild regularity conditions, the data integration process does not alter the underlying effect size, $\beta_{0}$, or target population defined by the original study design. Instead, the framework offers substantial gains in estimation efficiency without compromising validity. To maximize the utility of our proposed methods, careful selection of secondary outcomes prior to analysis is essential. We recommend that, at the study design stage, investigators identify secondary outcomes plausibly associated with the primary outcome, guided by prior literature or clinical expertise. During the analysis stage, a manageable number of secondary outcomes should be selected based on the sample size. A practical strategy is to prioritize secondary outcomes according to their strength of association with the primary outcome, for example, using Kendall’s tau correlation coefficient. Additional application guidance is provided in Section 6.

## 4 Simulation

We examined the numerical performance of our proposal under two main cases. In the first case, we considered a continuous primary outcome and one continuous secondary outcome. In the second case, we considered a continuous primary outcome and multiple continuous secondary outcomes. Besides, we examined extra cases: (1) the VIS estimator constructed with and without prior knowledge of zero coefficients; (2) a comparison of the PSI and PEPSI estimators to assess their equivalence when only one secondary outcome is present; (3) a binary primary outcome; (4) the secondary outcomes were mildly associated with the primary outcome; (5) the secondary outcomes with a lower signal-to-noise ratio; (6) prediction of the primary outcome from covariates and secondary outcomes using a machine learning model; and (7) small sample sizes. Details are presented in Section 3 of the online supplementary material. Ordinary least squares (OLS) regression and logistic regression were used for continuous and binary outcomes, respectively. For each case, we ran 10,000 Monte Carlo replications and reported the estimation bias, Monte Carlo standard deviation (MCSD), estimated standard error (SE), coverage probability of the 95% confidence interval (CP), and relative efficiency (RE; i.e. variance ratio compared with the naive estimator; larger than 1 implies efficiency gain).

### 4.1 Case 1: One continuous primary outcome and one continuous secondary outcome

We drew *n* i.i.d samples from the following model:


$$\left(\begin{array}{c} Y\\ {\tilde{Y}}_{1} \end{array}\right)=\left(\begin{array}{c} \beta_{0}\\ 0 \end{array}\right)+\left(\begin{array}{c} \beta^{T}\\ \theta_{1}^{T} \end{array}\right)\;X+\epsilon =\left(\begin{array}{c} 1\\ 0 \end{array}\right)+\left(\begin{array}{cccc} 1 & 1 & 1 & 1\\ 1 & 1 & 0 & 0 \end{array}\right)\;X+\epsilon ,$$

where *Y* and ${\tilde{Y}}_{1}$ are the primary and secondary outcomes, respectively; $\beta_{0}$ is the intercept, while $\beta =(\beta_{1},\ldots ,\beta_{4}{)}^{T}$ and $\theta_{1}=(\theta_{11},\ldots ,\theta_{14}{)}^{T}$ are the coefficient vectors. Some elements of $\theta_{1}$ were set to zero to facilitate data integration. As described in Section 3.1, null effects from certain covariates on outcomes are common in practice. However, it is important to note that our proposed methods do not require any prior knowledge to identify null-effect covariates, nor do they assume the primary parameters in β to be zero or non-zero. For illustration, we considered the case with $\beta =(1,\ldots ,1{)}^{T}$. The covariate vector $X=(X_{1},\ldots ,X_{4}{)}^{T}$ followed a multivariate normal distribution with mean 0, variance 1, and AR(1) correlation with parameter 0.5. The random noise term ϵ followed a bivariate normal distribution with mean 0, variance 1, and correlation coefficient *ρ*. We considered two sample sizes, n=300 and 600, and two correlation levels between *Y* and ${\tilde{Y}}_{1}$, ρ=0.5 or 0.8. For each configuration, we checked both the correctly specified model and a misspecified working model ($X_{1}$ was excluded from the working model) for the secondary analysis.

The results for ${\hat{\beta }}_{\mathrm{PSI}}$ are summarized in Table 1. Overall, the bias was negligible, the estimated SE was close to the MCSD, and the CP was near the nominal level, confirming that ${\hat{\beta }}_{\mathrm{PSI}}$ is consistent and that the asymptotic theory works well in finite samples. Compared with the naive estimator, ${\hat{\beta }}_{\mathrm{PSI}}$ was more efficient, i.e. smaller variance, especially for the coefficients ($\beta_{3}$ and $\beta_{4}$) whose corresponding coefficients in the secondary analysis were 0 ($\theta_{13}$ and $\theta_{14}$). The efficiency gain became more prominent as the correlation *ρ* increased, which was expected since the secondary outcome provided more information for the primary outcome. Moreover, ${\hat{\beta }}_{\mathrm{PSI}}$ remained unbiased and more efficient even under a misspecified working model, confirming the robustness of our proposal. More evaluations for ${\hat{\beta }}_{\mathrm{naive}}$ and ${\hat{\beta }}_{\mathrm{VIS}}$ with and without prior knowledge about zero coefficients are presented in Table S1 in the online supplementary material.

**Table 1.** Simulation results of Case 1

			Correctly specified model					Misspecified model				
			Bias	MCSD	SE	CP	RE	Bias	MCSD	SE	CP	RE
n=300	ρ=0.5	$\beta_{0}$	0.0	5.9	5.7	95	1.0	0.0	5.9	5.7	95	1.0
		$\beta_{1}$	0.0	6.7	6.6	95	1.0	0.2	6.7	6.6	95	1.0
		$\beta_{2}$	0.1	7.5	7.2	94	1.0	0.0	7.6	7.3	94	1.0
		$\beta_{3}$	− 0.1	6.7	6.4	94	1.3	0.0	7.0	6.9	95	1.2
		$\beta_{4}$	0.1	5.9	5.8	94	1.3	0.1	6.3	6.1	94	1.1
	ρ=0.8	$\beta_{0}$	0.0	5.9	5.7	94	1.0	0.0	5.9	5.7	94	1.0
		$\beta_{1}$	0.0	6.7	6.6	95	1.0	0.2	6.7	6.6	95	1.0
		$\beta_{2}$	0.1	7.2	6.9	94	1.1	− 0.1	7.4	7.1	94	1.1
		$\beta_{3}$	− 0.1	4.9	4.5	94	2.4	0.0	6.1	5.9	95	1.5
		$\beta_{4}$	0.1	4.4	4.1	94	2.3	0.1	5.5	5.3	94	1.5
n=600	ρ=0.5	$\beta_{0}$	0.0	4.1	4.1	95	1.0	0.0	4.1	4.1	95	1.0
		$\beta_{1}$	0.0	4.7	4.7	94	1.0	0.1	4.7	4.7	94	1.0
		$\beta_{2}$	0.0	5.2	5.1	95	1.1	− 0.1	5.2	5.2	95	1.0
		$\beta_{3}$	− 0.1	4.6	4.6	94	1.3	− 0.1	4.9	4.9	95	1.2
		$\beta_{4}$	0.1	4.1	4.1	95	1.3	0.0	4.4	4.4	94	1.1
	ρ=0.8	$\beta_{0}$	0.0	4.1	4.1	95	1.0	0.0	4.1	4.1	95	1.0
		$\beta_{1}$	0.0	4.7	4.7	95	1.0	0.1	4.8	4.7	95	1.0
		$\beta_{2}$	− 0.1	5.0	4.9	94	1.1	− 0.1	5.2	5.1	95	1.1
		$\beta_{3}$	− 0.1	3.3	3.2	94	2.6	− 0.1	4.3	4.2	95	1.5
		$\beta_{4}$	0.1	3.0	2.9	94	2.5	0.0	3.9	3.8	94	1.5

*Note*. All results except RE are multiplied by 100.

MCSD = Monte Carlo standard deviation; SE = estimated standard error; RE = relative efficiency over the naive estimator; CP = 95% coverage probability.

### 4.2 Case 2: One continuous primary outcome and multiple continuous secondary outcomes

Next, we generalized Case 1 by considering in total 50 secondary outcomes. We adopted the same data-generating process for the covariate X and the primary outcome *Y* as in Case 1. In addition to the secondary outcome ${\tilde{Y}}_{1}$ with ρ=0.8 in Case 1, we generated 49 more secondary outcomes. The model for the 50 secondary outcomes is $({\tilde{Y}}_{1},\ldots ,{\tilde{Y}}_{50}{)}^{T}=\Theta^{T}X+\epsilon$, where Θ is the coefficient matrix, with details specified in Section 3 of the online supplementary material. Briefly, the first 10 secondary outcomes had some zero entries in their coefficient vectors, while the remaining 40 outcomes had all non-zero entries. This setup mimics the real-world situation where only a subset of secondary outcomes have null effects for certain covariates. The random noise term ϵ followed a multivariate normal distribution with mean 0, variance 1, and constant correlation coefficient 0.5. The residual correlations between the primary outcome *Y* and the secondary outcomes $\left\{{\tilde{Y}}_{1},\ldots ,{\tilde{Y}}_{50}\right\}$ were 0.8,0.8,0.5,0.5,…,0.5, respectively. We considered two sample sizes, n=300 or 600, and two scenarios for integrating the secondary outcomes: (1) integrating 10 secondary outcomes $\left\{{\tilde{Y}}_{1},{\tilde{Y}}_{7},{\tilde{Y}}_{8},\ldots ,{\tilde{Y}}_{15}\right\}$; and (2) integrating 50 secondary outcomes $\left\{{\tilde{Y}}_{1},\ldots ,{\tilde{Y}}_{50}\right\}$. For each case, we evaluated and compared the performance of ${\hat{\beta }}_{\mathrm{PEPSI}}$ and ${\hat{\beta }}_{\mathrm{avg}}$. In particular, regarding ${\hat{\beta }}_{\mathrm{avg}}$, $\{w_{m}{\}}_{m=1}^{M}$ were determined by maximizing the sum of proportion of sample variance reduction (see Section 1 of the online supplementary material).

The results are summarized in Table 2. Both ${\hat{\beta }}_{\mathrm{PEPSI}}$ and ${\hat{\beta }}_{\mathrm{avg}}$ had negligible bias. As the sample size increased, the SE and CP approached the MCSD and the nominal 95% level, respectively, validating our asymptotic theory. Moreover, ${\hat{\beta }}_{\mathrm{PEPSI}}$ became more efficient as the number of secondary outcomes increased and showed a greater efficient gain compared with ${\hat{\beta }}_{\mathrm{avg}}$. Notably, the RE of ${\hat{\beta }}_{\mathrm{avg}}$ did not necessarily improve for certain parameters (e.g. $\beta_{3}$ and $\beta_{4}$) when the number of secondary outcomes increased from 10 to 50. This is expected because the averaging scheme makes some compromises among secondary outcomes, sacrificing some parameter-specific efficiency to maximize the overall proportion of sample variance reduction (C. Chen, Wang, et al., 2023), whereas the PEPSI integration scheme makes better use of secondary data (see Corollary 1). Similar conclusions were observed for all other cases with moderate sample sizes, as outlined in the online supplementary material (see online supplementary material Tables S3–S7 for cases 3–7). Besides, estimators ${\hat{\beta }}_{\mathrm{PSI}}$ and ${\hat{\beta }}_{\mathrm{PEPSI}}$ performed almost identically when only one secondary outcome was available (online supplementary material Table S2), further validating our theoretical findings.

**Table 2:** Simulation results of Case 2

			PEPSI					Averaging scheme				
			Bias	MCSD	SE	CP	RE	Bias	MCSD	SE	CP	RE
n=300	Integrating 10 outcomes	$\beta_{0}$	0.0	5.9	5.7	94	1.0	0.0	5.9	5.7	94	1.0
		$\beta_{1}$	0.0	5.7	5.3	93	1.4	0.0	6.5	6.3	94	1.1
		$\beta_{2}$	0.0	6.1	5.6	93	1.6	− 0.1	6.9	6.6	94	1.2
		$\beta_{3}$	0.0	4.7	4.3	93	2.6	0.0	4.9	4.6	94	2.3
		$\beta_{4}$	− 0.1	4.2	3.9	93	2.5	− 0.1	4.4	4.1	94	2.3
	Integrating 50 outcomes	$\beta_{0}$	0.1	6.0	5.6	93	0.9	0.1	5.8	5.7	94	1.0
		$\beta_{1}$	0.0	4.3	3.7	91	2.5	0.0	5.1	4.8	93	1.8
		$\beta_{2}$	0.0	4.5	4.0	91	2.7	0.0	5.3	5.0	94	2.0
		$\beta_{3}$	0.0	4.6	4.0	91	2.7	− 0.1	5.4	5.0	93	2.0
		$\beta_{4}$	0.0	4.3	3.7	91	2.5	0.0	5.1	4.8	94	1.8
n=600	Integrating 10 outcomes	$\beta_{0}$	0.0	4.1	4.0	95	1.0	0.0	4.1	4.1	95	1.0
		$\beta_{1}$	0.0	3.9	3.8	94	1.4	0.0	4.5	4.5	95	1.1
		$\beta_{2}$	0.1	4.2	4.0	94	1.6	0.1	4.8	4.7	95	1.2
		$\beta_{3}$	0.0	3.2	3.1	94	2.7	0.0	3.4	3.3	94	2.4
		$\beta_{4}$	0.0	2.9	2.7	94	2.7	0.0	3.0	2.9	94	2.4
	Integrating 50 outcomes	$\beta_{0}$	0.0	4.2	4.0	94	1.0	0.0	4.1	4.1	95	1.0
		$\beta_{1}$	0.0	2.9	2.7	93	2.7	0.0	3.5	3.4	94	1.8
		$\beta_{2}$	0.0	3.1	2.8	93	3.1	0.0	3.8	3.6	94	2.1
		$\beta_{3}$	0.0	3.1	2.8	93	3.1	0.0	3.7	3.6	94	2.1
		$\beta_{4}$	0.1	2.9	2.7	93	2.7	0.1	3.5	3.4	94	1.8

*Note*. All results except RE are multiplied by 100.

MCSD = Monte Carlo standard deviation; SE = estimated standard error; RE = relative efficiency over the naive estimator; CP = 95% coverage probability.

**Figure 2.**
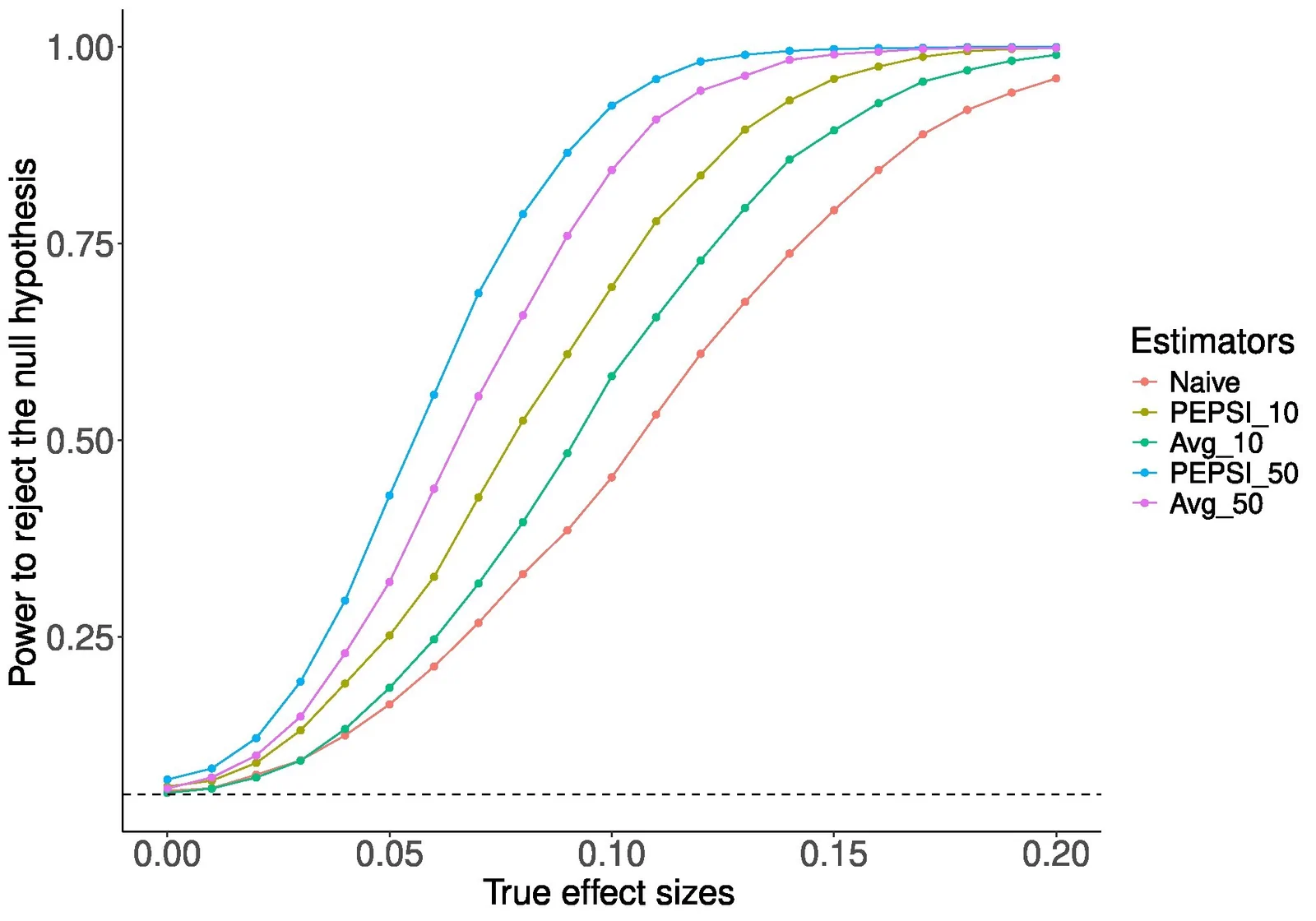
Statistical power evaluation of various estimators for rejecting the null hypothesis $H_{0}\;:\;\beta_{2}=0$ under different true values of $\beta_{2}$. Naive: the standard estimator without data integration; PEPSI_10 (50) and Avg_10 (50): the PEPSI and averaging scheme estimators integrating 10 (50) secondary outcomes, respectively.

To evaluate the benefit of data integration in hypothesis testing, we considered a modified Case 2 by varying the effect size $\beta_{2}$ from 0 to 0.2. Using the asymptotic normality of ${\hat{\beta }}_{\mathrm{naive}}$, ${\hat{\beta }}_{\mathrm{PEPSI}}$, and ${\hat{\beta }}_{\mathrm{avg}}$, we constructed Wald tests and compared their rejection rates for the null hypothesis $H_{0}\;:\;\beta_{2}=0$ with a 0.05 significance levels. The results are summarized in Figure 2. Notable, both ${\hat{\beta }}_{\mathrm{PEPSI}}$ and ${\hat{\beta }}_{\mathrm{avg}}$ maintained type I error rates near 0.05 under the null, while achieving greater power than ${\hat{\beta }}_{\mathrm{naive}}$ under the alternative, especially as more secondary outcomes were integrated. Overall, ${\hat{\beta }}_{\mathrm{PEPSI}}$ was more powerful than ${\hat{\beta }}_{\mathrm{avg}}$ with moderate sample sizes.

**Figure 3.**
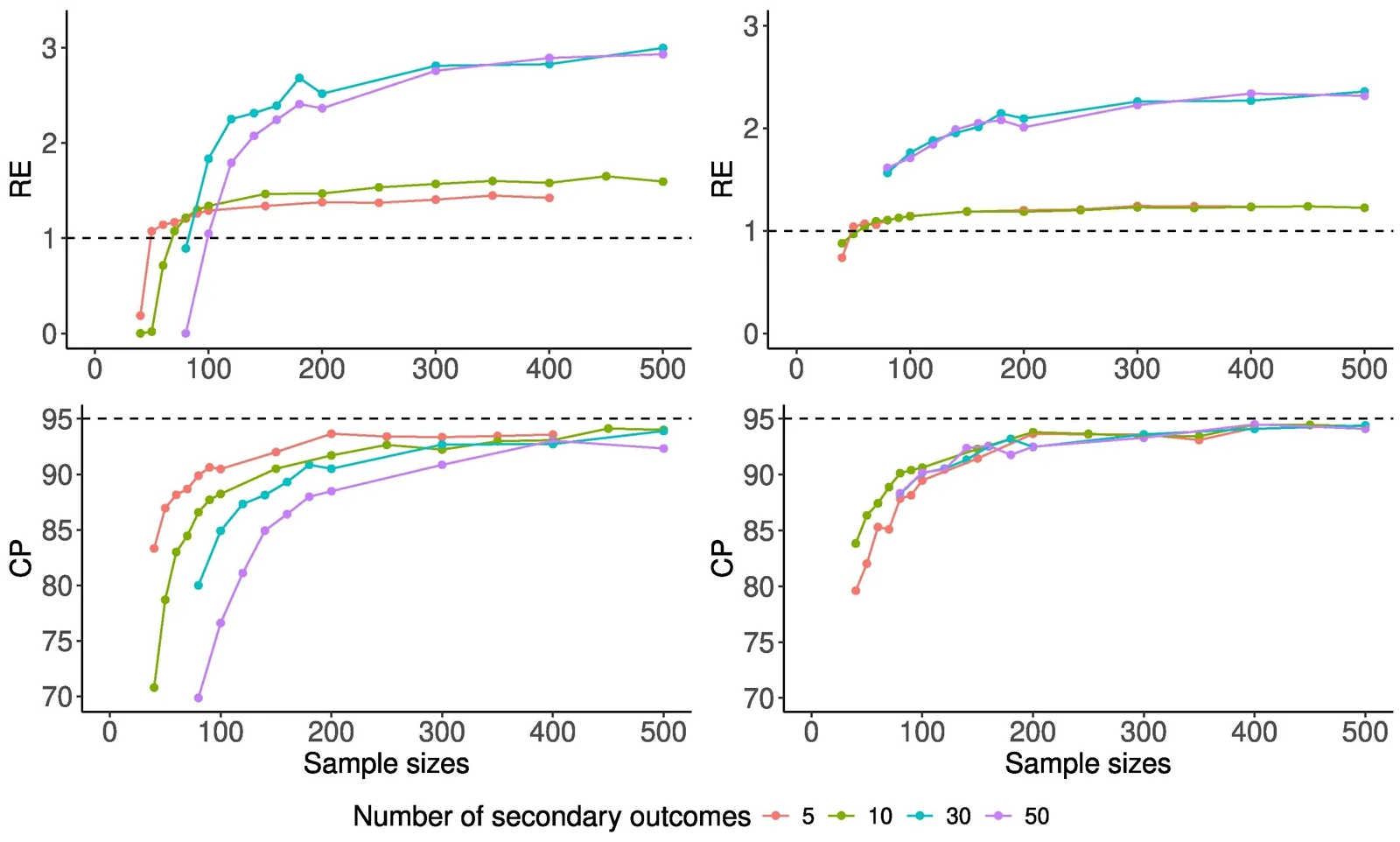
Relative efficiency (RE) and 95% coverage probabilities (CP) of the PEPSI estimator (left) and the averaging scheme estimator (right) for estimating $\beta_{2}$ across varying sample sizes and numbers of secondary outcomes.

Moreover, we conducted additional simulations to evaluate the efficiency gain and asymptotic inference accuracy across a range of sample sizes. Building on the two secondary outcomes settings in Case 2 (i.e. 10 and 50 outcomes), we added two more scenarios: (1) 5 secondary outcomes $\left\{{\tilde{Y}}_{1},{\tilde{Y}}_{7},{\tilde{Y}}_{11},{\tilde{Y}}_{12},{\tilde{Y}}_{13}\right\}$ and (2) 30 secondary outcomes $\left\{{\tilde{Y}}_{1},{\tilde{Y}}_{2},{\tilde{Y}}_{6},{\tilde{Y}}_{7},\ldots ,{\tilde{Y}}_{33}\right\}$. For each scenario, we examined a range of sample sizes to evaluate the empirical efficiency gain and CP of ${\hat{\beta }}_{\mathrm{PEPSI}}$ and ${\hat{\beta }}_{\mathrm{avg}}$. For simplicity and clarity, we only reported the results for the $\beta_{2}$ estimators, as similar patterns were observed for other coefficients. As shown in Figure 3, ${\hat{\beta }}_{\mathrm{PEPSI}}$ outperformed ${\hat{\beta }}_{\mathrm{naive}}$ in efficiency once the sample size exceeded a certain threshold (e.g. 75), and its coverage probability approached the nominal level as the sample size increased. However, integrating more secondary outcomes required a larger sample size to achieve efficiency gain and reliable coverage, due to the increased DoF. Similar trends were observed for ${\hat{\beta }}_{\mathrm{avg}}$; however, it achieved more stable performance with smaller sample sizes and maintained coverage closer to the nominal level, suggesting greater robustness in small/moderate sample size settings (e.g. n=100 to 300). Further evaluations are provided in Section 3 of the online supplementary material.

## 5 Application

In this case study, we investigated the effect of cigarette smoking on liver health and explored how it differed across age groups using the UKB data. Specifically, we considered liver PDFF and LBRS as two primary outcomes, with past smoking habits as the primary exposure. Participants were categorized into ‘heavy smokers’ (those who smoked most or all days) and ‘others’. It was also of significant interest to study whether the effect of smoking differed between older and younger adults, given evidence that older smokers might have an increased risk of becoming frail (Kojima et al., 2018). Moreover, we aimed to evaluate: (1) the validity of the predicted LBRS by comparing its performance with analysis using liver PDFF as the primary outcome, and (2) the utility of PEPSI for integrating information from secondary outcomes defined from both Type 1 (PDFF) and Type 3 (LBRS) sources.

### 5.1 The construction of LBRS

**Figure 4.**
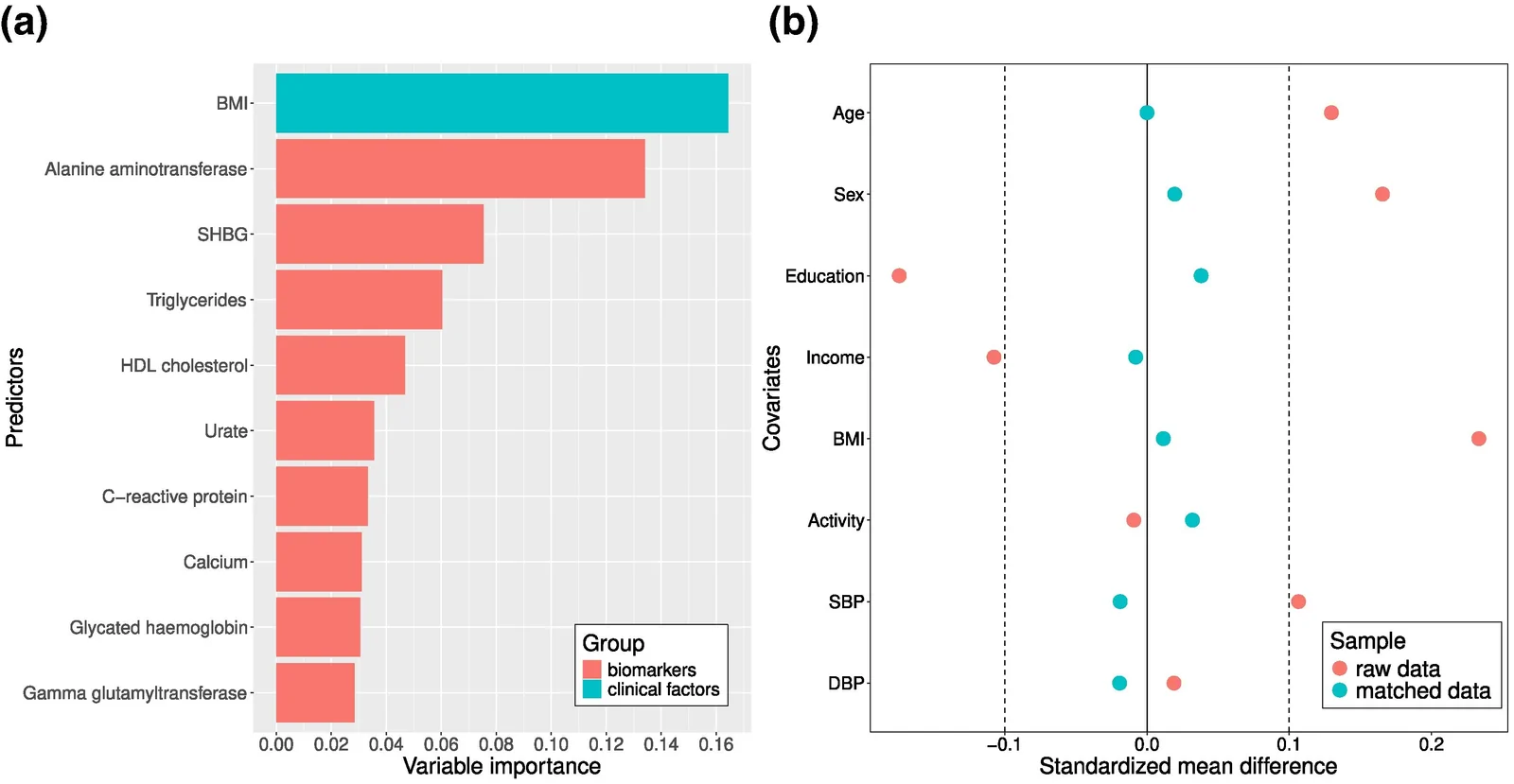
(a) The variable importance plot with the top 10 predictors contributing most to liver PDFF prediction. (b) The love plot assessing covariate balances of the raw and matched data.

To derive LBRS, we predicted liver PDFF values by building a machine learning model using clinical factors, and the blood and urine biomarkers listed in the online supplementary material, as predictors. Other factors were used, including a variety of baseline characteristics, such as smoking status (heavy smokers vs. others), age (≥ 65 vs. others), sex (male vs. female), education level (college or university degree vs. others), income level (≥ £31,000 annually vs. others), BMI, sum of days performing walking, systolic blood pressure, and diastolic blood pressure, which comprehensively captured participants’ demographics, lifestyle, and health conditions. The training data consisted of a cohort with complete records of liver PDFF, biomarkers and other factors, with a sample size of 2,225. We applied XGBoost and tuned hyperparameters to minimize the mean squared error via fivefold cross-validation. The hyperparameters included learning rates, depths of trees, the minimum number of instances in a child node, and the proportions of samples and variables in a tree. The resulting prediction model explained 26% of the variability in liver PDFF variable in out of bag samples. The top 10 important biomarkers for liver PDFF prediction are presented in Figure 4a, where BMI and alanine aminotransferase (ALT) emerged as the two most influential factors. Recent findings from another study showed that long-term high-normal ALT levels increased the risk of developing metabolic dysfunction-associated fatty liver disease (MAFLD) (J.-F. Chen et al., 2024), supporting the external validity of our prediction model.

### 5.2 Facilitating casual interpretation

We excluded the training cohort from the downstream analysis. To unbiasedly assess the smoking effect while accounting for potential confounding factors, we applied propensity-score matching (Rosenbaum & Rubin, 1983 ) to align the basic profiles between ‘heavy smokers’ and ‘others’. Although matching is not required to implement our method, however, it facilitates the estimation of exposure effects within the region of common support, thereby mitigating extrapolation to areas where exposed and unexposed groups differ substantially and thus leading more unbiased comparison. To study the modifying effect of age on smoking, as suggested in the literature (Jang et al., 2023), we conducted exact matching on age group and applied propensity score matching on the remaining covariates, with propensity scores estimated using logistic regression. After matching, we obtained 1,893 subjects in each group of ‘heavy smokers’ and ‘others’, with 239 (12.6%) older adults in each group. This matching process yielded more balanced baseline covariate distributions compared to the original, unmatched cohort (Figure 4b). The profile of the matched cohort for the final analysis are summarized in Table S8 in the online supplementary material.

After matching, we regressed LBRS on smoking status, age, and their interaction, and then calculated the marginal effect of smoking on LBRS, as well as the subgroup effects for different age groups. We applied the PEPSI framework for parameter estimation by integrating information from the secondary outcomes (i.e. blood and urine biomarkers). OLS regression was used for both the primary and secondary data analysis, and we compared the PEPSI estimator with a naive OLS estimator that did not integrate information from secondary data as a benchmark. For both estimators, we calculated 95% confidence intervals and *p*-values from Wald tests against the null hypothesis that the underlying values were zeros. In addition, we considered E-value to explore the potential impact of unmeasured confounders (VanderWeele & Ding, 2017), such as dietary preference.

### 5.3 Results interpretation

The results are summarized in Table 3. Compared with the naive OLS estimator, the PEPSI estimator yielded similar point estimates, implying that the proposed integrative learning does not introduce estimation bias to the original analysis. However, the PEPSI estimator showed substantial variance reduction and narrower confidence intervals, with RE greater than 4 for many estimates. This signifies the utility of the PEPSI estimator when integrating information from secondary outcomes. Notably, the PEPSI estimator identified more statistically significant factors, whereas the naive OLS estimator failed to detect any significant association. In particular, the PEPSI results showed that the LBRS was significantly lower in older adults compared to younger adults when controlling for the same smoking habit. Regarding cigarette smoking, we observed significantly higher LBRS values in heavy smokers. A more detailed analysis revealed that cigarette smoking had a positive and significant effect on LBRS in older adults, and this effect was significantly larger than that in younger adults. In contrast, cigarette smoking might not have a significant impact on LBRS among younger adults. These findings suggest that cigarette smoking affects LBRS, with heterogeneous effects across different sub-populations. Furthermore, the E-values for the smoking effects were 1.45 for adults aged 65 or older and 1.21 for the general population. These values imply that our analysis exhibit moderate/mild robustness against unmeasured confounders (VanderWeele & Ding, 2017).

**Table 3.** Results of the naive OLS and PEPSI methods for estimating the smoking effect on LBRS

		OLS					PEPSI					
		EST	SE	LL	UL	P	EST	SE	LL	UL	P	RE
Regression parameters	Intercept	5.526	0.077	5.376	5.677	0.000	5.499	0.057	5.387	5.611	0.000	1.813
	Smoking	0.055	0.106	− 0.154	0.264	0.605	0.063	0.052	− 0.040	0.166	0.229	4.138
	Smoking:Age	0.084	0.267	− 0.438	0.607	0.752	0.281	0.118	0.049	0.513	0.018	5.087
	Age	− 0.211	0.187	− 0.579	0.156	0.259	− 0.227	0.093	− 0.410	− 0.044	0.015	4.030
Effect of smoking	Age below 65	0.055	0.106	− 0.154	0.264	0.605	0.063	0.052	− 0.040	0.166	0.229	4.138
	Age no less than 65	0.139	0.245	− 0.340	0.619	0.569	0.344	0.108	0.132	0.555	0.001	5.137
	Overall	0.066	0.098	− 0.126	0.258	0.503	0.098	0.048	0.004	0.193	0.041	4.140

*Note*. EST = parameter estimates; SE = estimated standard errors; LL & UL = 95% lower and upper confidence limits of estimates; P = *p*-values; RE = relative efficiency, ratios of estimated variances between naive OLS and PEPSI model estimates.

### 5.4 Results validation

We validated the findings by repeating the analysis using liver PDFF as the primary outcome, while keeping all other procedures identical to those previously described. A total of 1,078 subjects were included in this analysis (demographics were similar to the primary analysis, see Table S9 in the online supplementary material), and the results are summarized in Table 4. Most estimates showed consistent patterns with Table 3, such as significant smoking effects in older adults but not in younger individuals. These findings validate our findings above and suggest that LBRS can serve as a reliable surrogate outcome for the raw liver PDFF data. However, the estimated effect sizes were larger when using liver PDFF. Regarding efficiency gain, the RE was less pronounced than in the analysis using LBRS, which is expected given the weaker associations between the primary outcome (liver PDFF) and the secondary outcomes. Nevertheless, the PEPSI estimates were still more efficient than the OLS estimates, with some RE values reaching as high as 1.6. Additionally, we conducted two supplementary analysis: one in which liver PDFF values were predicted only by the blood and urine biomarkers, and another external validation analysis using the Alzheimer’s Disease Neuroimaging Initiative dataset (Mueller et al., 2005). Both analysis yielded conclusions consistent with our primary findings and are presented in Tables S10 and S14 in the online supplementary material.

**Table 4.** Analysis results of the naive OLS and PEPSI methods for estimating the smoking effect on raw liver PDFF

		OLS					PEPSI					
		EST	SE	LL	UL	P	EST	SE	LL	UL	P	RE
Regression parameters	Intercept	5.706	0.301	5.117	6.295	0.000	5.642	0.285	5.083	6.201	0.000	1.111
	Smoking	− 0.195	0.413	− 1.003	0.614	0.637	− 0.157	0.376	− 0.894	0.581	0.678	1.202
	Smoking:Age	2.537	0.977	0.621	4.452	0.009	1.829	0.800	0.261	3.398	0.022	1.491
	Age	− 1.071	0.534	− 2.118	− 0.024	0.045	− 0.785	0.470	− 1.706	0.137	0.095	1.291
Effect of smoking	Age below 65	− 0.195	0.413	− 1.003	0.614	0.637	− 0.157	0.376	− 0.894	0.581	0.678	1.202
	Age no less than 65	2.342	0.886	0.606	4.078	0.008	1.673	0.709	0.284	3.062	0.018	1.561
	Overall	0.187	0.375	− 0.549	0.922	0.619	0.118	0.338	− 0.544	0.781	0.726	1.232

*Note*. EST = parameter estimates; SE = estimated standard errors; LL & UL = 95% lower and upper confidence limits of estimates; P = *p*-values; RE = relative efficiency, ratios of estimated variances between naive OLS and PEPSI model estimates.

## 6 Discussion

In this article, we have developed a new integrative learning framework that enhances inferences on the primary outcome by leveraging additional information from multiple cross-sectional secondary outcomes. The simulation/example code is available online (github.com/daxuand1/pepsi). In our case study using UKB data, this approach significantly improved the analysis, revealing that cigarette smoking may adversely affect liver health, with particularly stronger effects observed in older adults. Notably, our findings suggest that older adult smokers may have a higher risk of developing fatty liver, while younger smokers may not experience the same impact. This discrepancy could be due to older adults having longer smoking histories or multiple pathways, including toxic, immunologic, and oncogenic factors, making them more susceptible as their biological systems become less resilient with age (Kojima et al., 2018). These results enrich the current knowledge about the detrimental effects of cigarette smoking on liver health (Marti-Aguado et al., 2022) and support public health recommendations for older adults to quit smoking. Furthermore, by comparing the results obtained from the predicted LBRS with those from the original liver PDFF measure, we observed reduced covariate effects, indicating that the predicted risk score may lose some information from the original outcome, leading to decreased statistical power for detecting covariate significance. Thus, integrating information from individual predictors as secondary outcomes is a natural and promising approach to enhance the statistical performance of risk prediction score analysis. Additionally, the efficiency gain was substantially larger for the PEPSI estimator when the predicted LBRS serves as the primary outcome compared to the original liver PDFF outcome. This finding implies that, in practice, the PEPSI estimator is especially favourable and strongly recommended when secondary outcomes are obtained from Type 2 or 3 sources.

Extensive simulation studies suggest that the proposed methods perform well when $n\geq 40p_{0}+40K$ and $n\geq 40p_{0}+20K$ for ${\hat{\beta }}_{\mathrm{PEPSI}}$ and ${\hat{\beta }}_{\mathrm{avg}}$, respectively, where $p_{0}$ is the number of parameters in the primary model, and *K* is the extra DoF in the secondary models (defined in Theorem 2). These observations provide practical guidance for study design. Moreover, our simulations suggest that for sample sizes around 300 or fewer, no more than 10 secondary outcomes should be used with ${\hat{\beta }}_{\mathrm{PEPSI}}$; for sample sizes between 300 and 500, up to 30 secondary outcomes can be integrated; and for sample sizes of 500 or above, ${\hat{\beta }}_{\mathrm{PEPSI}}$ can accommodate 50 secondary outcomes. Otherwise, ${\hat{\beta }}_{\mathrm{avg}}$ is preferred over ${\hat{\beta }}_{\mathrm{PEPSI}}$. Further evaluations are needed to develop more comprehensive guidelines for practical use.

From a methodological standpoint, there are several potential extensions of the PEPSI framework. First, while this article focuses on low-dimensional covariates as an initial study of PEPSI, it would be valuable to explore extensions to high-dimensional settings. Second, the current framework is based on the generalized linear model, which could be adapted for use with missing data, time-to-event models, or causal inference. Third, beyond integrating data within a single study, adapting the PEPSI framework for information integration across multiple independent datasets holds great promise. Fourth, it would be interesting to develop an empirical likelihood ratio test within the proposed framework. In theory, it may be possible to develop an ELR test by stacking the estimating equations from both the primary and secondary outcomes and jointly estimating the parameters under a penalized empirical likelihood framework (Leng & Tang, 2012). However, as discussed in Section 3.2, this approach may suffer from high-dimensional estimating equations and can be computationally intensive and unstable. These extensions require considerable effort and warrant further research.

Despite its methodological advantages, we acknowledge two theoretical challenges that remain unaddressed in this article. First, although we have provided a useful guideline for selecting secondary outcomes (Section 3.2), quantifying uncertainty associated with this selection process is necessary but remains challenging. Nonetheless, it is important to emphasize that the selection of secondary outcomes does not alter the target population and the underlying effect size defined by the primary outcome and original study design. Second, when applying the PEPSI estimator with a very large number of secondary outcomes (e.g. thousands), the standard errors may be under-estimated. This issue can be partially attributed to uncertainties arising from implementing PCA and tuning parameter selection in numerous penalized optimizations. Addressing these challenges requires more advanced theoretical development, which is beyond the scope of this article.

## Supplementary Material

qkaf081_Supplementary_Data

## Data Availability

The UK Biobank data are publicly available to approved researchers through the UK Biobank Access Management System (https://www.ukbiobank.ac.uk). The ADNI data are publicly accessible through the Alzheimer’s Disease Neuroimaging Initiative data repository (https://adni.loni.usc.edu), subject to standard data-use agreements.
