# Supplementary material for: A new integrative learning framework for integrating multiple secondary outcomes into primary outcome analysis: a case study on liver health
Source: J R Stat Soc Series B Stat Methodol. 2025 Dec 30;88(4):1160–80. doi: 10.1093/jrsssb/qkaf081 (PMC13579266; doi:10.1093/jrsssb/qkaf081)
Supplement: Supplementary file 1 [file qkaf081_supplementary_data.pdf]

Supplementary Materials of "A New Integrative Learning Framework for Integrating Multiple Secondary Outcomes into Primary Outcome Analysis: A Case Study on Liver Health"

Daxuan Deng[1], Peisong Han[2], Shuo Chen[3], Ming Wang[4], Chixiang Chen[3]
[1] Department of Public Health Sciences,
Penn State College of Medicine, Hershey, PA, USA
[2] Biostatistics Innovation Group, Gilead Sciences, Foster City, CA, USA
[3] Department of Epidemiology and Public Health,
University of Maryland School of Medicine, Baltimore, MD, USA
[4] Department of Population and Quantitative Health Sciences,
Case Western Reserve University, Cleveland, OH, USA

December 11, 2025

This document includes additional theoretical results outlined in the manuscript (Section 1); regularity conditions and technical proofs for theorems and corollaries (Section 2); further simulations discussed in the manuscript (Section 3); and additional results for data application (Section 4).

1 More theoretical investigations

In this section, we investigate deeper on theoretical properties of our proposed estimators and present some corollaries (e.g, the optimal use of secondary outcomes). Most of mathematical notations have been defined in Section 3 of the manuscript. We will thus not repeat their definitions in this document. Corresponding regularity conditions and proofs

are demonstrated in the next section.

1.1 PSI estimator

The following corollary explicitly justifies the use of penalty function.

Corollary S1 *Given a working estimating function* $\boldsymbol{g}_m(\tilde{\boldsymbol{D}}_m; \boldsymbol{\theta}_m)$, $\hat{\boldsymbol{\beta}}_{PSI}$ *is less efficient if any penalty term on* $\boldsymbol{\theta}^*_{m(1)}$, *that is* $\rho_{\tau_m}(|\theta_{mj}|)$, $j = 1, 2, \ldots, q_m$, *is dropped.*

Since Theorem 1 in the manuscript shows that $\hat{\boldsymbol{\theta}}_{m(1)} = \mathbf{0}$ with probability tending to 1, it is equivalent to say that identifying more zero entries in $\boldsymbol{\theta}^*_m$ leads to more efficient estimate of $\boldsymbol{\beta}$ asymptotically. In the extreme case where we fail to identify any zero entry of $\boldsymbol{\theta}_m$, then $\hat{\boldsymbol{\beta}}_{PSI}$ will reduce to $\hat{\boldsymbol{\beta}}_{EIS}$.

The following corollary characterized the optimality of $\hat{\boldsymbol{\beta}}_{PSI}$ in integrating extra information.

Corollary S2 *Given a working estimating function* $\boldsymbol{g}_m(\tilde{\boldsymbol{D}}_m; \boldsymbol{\theta}_m)$ *and a penalized empirical likelihood estimate* $\hat{\boldsymbol{\theta}}$ *from (2) in the manuscript,* $\hat{\boldsymbol{\beta}}_{PSI}$ *is the most efficient estimator among the following class of estimating equations*

$$\left\{ \frac{1}{n} \sum_{i=1}^{n} \left\{ \boldsymbol{f}(\boldsymbol{D}_i; \boldsymbol{\beta}) + \boldsymbol{F}_m \boldsymbol{g}_m(\tilde{\boldsymbol{D}}_{mi}; \hat{\boldsymbol{\theta}}_m) \right\} = o_p(n^{-1/2}) \right\},$$

where $\boldsymbol{F}_m$ *is an arbitrary conformable matrix.*

Combining Corollary S2 and Corollary 1 in the main manuscript, we conclude that $\hat{\boldsymbol{\beta}}_{PSI}$ and $\hat{\boldsymbol{\beta}}_{PEPSI}$ are equivalent when there is only one secondary outcome to integrate. Hence $\hat{\boldsymbol{\beta}}_{PEPSI}$ can be viewed as a generalization of $\hat{\boldsymbol{\beta}}_{PSI}$.

1.2 Averaging scheme estimator

This section provides additional details on the proposed Averaging scheme, with the asymptotic properties of $\hat{\boldsymbol{\beta}}_{avg}$ summarized below.

Theorem S1 *Under mild conditions listed in Section 2, $\hat{\boldsymbol{\beta}}_{avg}$ is consistent to $\boldsymbol{\beta}_0$, and is asymptotic normal with an asymptotic variance*

$$\boldsymbol{V}_{avg} = \boldsymbol{\Gamma}^{-1}\left\{\boldsymbol{\Sigma} - \sum_{m=1}^{M} w_m^2 \boldsymbol{\Lambda}_m(\boldsymbol{S}_m + \boldsymbol{P}_m)\boldsymbol{\Lambda}_m^T + \sum_{i \neq j}^{M} w_i w_j \boldsymbol{\Lambda}_i(\boldsymbol{S}_i + \boldsymbol{P}_i)\tilde{\boldsymbol{A}}_{ij}(\boldsymbol{S}_j + \boldsymbol{P}_j)\boldsymbol{\Lambda}_j^T\right\}\boldsymbol{\Gamma}^{-1,T},$$

where $\boldsymbol{\Gamma}$, $\boldsymbol{\Sigma}$, $\boldsymbol{\Lambda}_m$, $\boldsymbol{S}_m$, and $\boldsymbol{P}_m$ are defined in Theorem 1 in the main manuscript; $\tilde{\boldsymbol{A}}_{ij} = E\{\boldsymbol{g}_i(\tilde{\boldsymbol{D}}_i;\boldsymbol{\theta}_i^*)\boldsymbol{g}_j^T(\tilde{\boldsymbol{D}}_j;\boldsymbol{\theta}_j^*)\}$.

Recall that $\boldsymbol{V}_{naive} = \boldsymbol{\Gamma}^{-1}\boldsymbol{\Sigma}\boldsymbol{\Gamma}^{-1,T}$; thus, $\hat{\boldsymbol{\beta}}_{avg}$ is more efficient than $\hat{\boldsymbol{\beta}}_{naive}$. However, the degree of efficiency gain depends crucially on the choice of weights $\{w_m\}_{m=1}^M$. These weights can be determined by clinicians based on their scientific insights, or alternatively, determined using a data-driven approach by maximizing some pre-specified objective functions. For example, if the primary interest lies in the first coefficient, we can define the objective function as $F_{obj} = (1, 0, \ldots, 0)\boldsymbol{V}_{avg}(1, 0, \ldots, 0)^T$. On the other hand, if global efficiency improvement is of primary interest, we may instead maximize the overall proportion of variance reduction across all coefficients. In this case, the objective function can be defined as $F_{obj} = \text{trace}[(\boldsymbol{V}_{naive} - \boldsymbol{V}_{avg})\{\text{diag}(\boldsymbol{V}_{null})\}^{-1}]$, where $\text{diag}(\boldsymbol{A})$ denotes a diagonal matrix constructed from the diagonal elements of $\boldsymbol{A}$. In practice, we do not know $\boldsymbol{V}_{naive}$ or $\boldsymbol{V}_{avg}$ in advance, so we can plug-in their consistent estimates, which can be calculated using a naive estimate of $\boldsymbol{\beta}$ without data integration.

2 Conditions and proofs

To proceed, we simplify some notation hereafter. We omit the data variables ($\boldsymbol{D}$ and $\tilde{\boldsymbol{D}}$) in functions and use the subscript i to distinguish subjects. For example, the primary estimating function for the subject i is expressed as $\boldsymbol{f}_i(\boldsymbol{\beta})$. We further compress parameters when functions are evaluated at the true values, e.g., $\boldsymbol{f}_i \equiv \boldsymbol{f}_i(\boldsymbol{\beta}_0)$.

2.1 Conditions

We assume the following conditions hold for the primary model and every secondary model $(1 \leqslant m \leqslant M)$.

Condition 1 $\boldsymbol{\Sigma}$ *is positive definite,* $\boldsymbol{f}(\boldsymbol{\beta})$ *has a continuous second derivative in a neighborhood of* $\boldsymbol{\beta}_0$*, and* $\boldsymbol{\Gamma}$ *is not singular. In addition,* $\|\partial \boldsymbol{f}(\boldsymbol{\beta})/\partial \boldsymbol{\beta}^T\|$, $\|\partial^2 \boldsymbol{f}(\boldsymbol{\beta})/\partial \boldsymbol{\beta}^2\|$ *are bounded by some integrable functions around* $\boldsymbol{\beta}_0$*.*

Condition 2 $\boldsymbol{A}_m$ *is positive definite,* $\boldsymbol{g}_m(\boldsymbol{\theta}_m)$ *has a continuous second derivative in a neighborhood of* $\boldsymbol{\theta}_m^*$*, and* $\boldsymbol{B}_m$ *has rank* p_m*. In addition,* $\|\partial \boldsymbol{g}_m(\boldsymbol{\theta}_m)/\partial \boldsymbol{\theta}_m^T\|$, $\|\partial^2 \boldsymbol{g}_m(\boldsymbol{\theta}_m)/\partial \boldsymbol{\theta}_m^2\|$*, and* $\|\boldsymbol{g}_m(\boldsymbol{\theta}_m)\|^3$ *are bounded by some integrable functions around* $\boldsymbol{\theta}_m^*$*.*

Condition 3 *As* $n \to \infty$*,* $\tau_m n^{1/2} \to \infty$*, and* $\min_{q_m+1 \leqslant j \leqslant p_m} |\theta_{mj}^*|/\tau_m \to \infty$*.*

Condition 4 $\max_{q_m+1 \leqslant j \leqslant p_m} \rho'_{\tau_m}(|\theta_{mj}^*|) = o(n^{-1/2})$*, and* $\max_{q_m+1 \leqslant j \leqslant p_m} \rho''_{\tau_m}(|\theta_{mj}^*|) = o(1)$*.*

$\boldsymbol{\Sigma}$, $\boldsymbol{\Gamma}$, $\boldsymbol{A}_m$ and $\boldsymbol{B}_m$ are defined in Theorem 1 in the main manuscript. Conditions 1 and 2 are regularities widely adopted in generalized method of moments [Newey and McFadden, 1994] and empirical likelihood [Qin and Lawless, 1994] to ensure asymptotic expansions. Conditions 3 and 4 are regularities on the penalty. Condition 3 states that the nonzero parameters can not converge to zero too fast, instead, it should dominate the magnitude of

tuning parameter, a commonly used assumption in literature [Tang and Leng, 2010, Leng and Tang, 2012]. Condition 4 controls the impact of penalty on the non-zero component, which holds by many penalty functions such as the SCAD penalty [Fan and Li, 2001].

2.2 Proof of Theorem 1

We sketch the proof of Theorem 1, which consists of three steps: first, prove that $P(\hat{\boldsymbol{\theta}}_{m(1)} = \mathbf{0}) \to 1$. Here, the notation $\to$ denotes convergence as sample size increases; second, derive the asymptotics of penalized empirical likelihood estimators; third, derive the asymptotic property of $\hat{\boldsymbol{\beta}}_{PSI}$. Steps 1 and 2 are partially based on Leng and Tang [2012] focusing on the high dimensional variable selection setting. Here, we consider the case where the dimension of considered covariates are fixed, which well aligns with our data application. Step 3 derives the asymptotic property of $\hat{\boldsymbol{\beta}}_{PSI}$.

Step 1. By empirical likelihood theory [Qin and Lawless, 1994, Owen, 2001], we have $\|\hat{\boldsymbol{\theta}}_m - \boldsymbol{\theta}^*_m\| = O_p(n^{-1/2})$, $\|\hat{\boldsymbol{\lambda}}_m\| = O_p(n^{-1/2})$. To facilitate the proof, we focus on the parameter values of $(\boldsymbol{\theta}_m^T, \boldsymbol{\lambda}_m^T)^T$ within a $n^{-1/2}$-neighbourhood of $(\boldsymbol{\theta}_m^{*T}, \mathbf{0}^T)^T$. In this neighbourhood, we have $\boldsymbol{\lambda}_m^T(\boldsymbol{\theta}_m)\boldsymbol{g}(\boldsymbol{\theta}_m) = o_p(1)$ uniformly hold. For $1 \leqslant j \leqslant p_m$, we have

$$
\begin{aligned}
\frac{1}{n}\frac{\partial l_{pm}(\boldsymbol{\theta}_m)}{\partial \theta_{mj}} &= \frac{1}{n}\sum_{i=1}^{n} \boldsymbol{\lambda}_m^T \frac{\partial \boldsymbol{g}_{mi}(\boldsymbol{\theta}_m)}{\partial \theta_{mj}}\{1 + o_p(1)\} + \rho'_{\tau_m}(|\theta_{mj}|)\mathrm{sign}(\theta_{mj}) \\
&= \rho'_{\tau_m}(|\theta_{mj}|)\mathrm{sign}(\theta_{mj}) + O_p(n^{-1/2}).
\end{aligned}
$$

By Condition 3, $\tau_m n^{1/2} \to \infty$, and thus $\|\boldsymbol{\theta}_{m(1)} - \boldsymbol{\theta}^*_{m(1)}\| = \|\boldsymbol{\theta}_{m(1)}\| = O_p(n^{-1/2}) = o_p(\tau_m)$. In this case, $\rho'_{\tau_m}(|\theta_{mj}|)\mathrm{sign}(\theta_{mj})$ dominates the sign of $\partial l_{pm}(\boldsymbol{\theta}_m)/\partial \theta_{mj}$ for $j = 1, 2, \ldots, q_m$ as

$n \to \infty$. With probability tending to one, we have

$$\frac{\partial l_{pm}(\boldsymbol{\theta}_m)}{\partial \theta_{mj}} < 0, \text{ for } \theta_{mj} \in (-\epsilon_n, 0) \text{ and } \frac{\partial l_{pm}(\boldsymbol{\theta}_m)}{\partial \theta_{mj}} > 0, \text{ for } \theta_{mj} \in (0, \epsilon_n),$$

where $\epsilon_n = cn^{-1/2}$ for some constant c. According to Fan and Li [2001], it implies that $\hat{\theta}_{mj} = 0$, for $j = 1, 2, \ldots, q_m$. Therefore, we conclude that $P(\hat{\boldsymbol{\theta}}_{m(1)} = \mathbf{0}) \to 1$.

Step 2. Given that $P(\hat{\boldsymbol{\theta}}_{m(1)} = \mathbf{0}) \to 1$, minimizing the penalized loss (2) in the manuscript is equivalent to minimizing the following loss function:

$$\sum_{i=1}^{n} \log\{1 + \boldsymbol{\lambda}_m^T(\boldsymbol{\theta}_m)\boldsymbol{g}_m(\tilde{\boldsymbol{D}}_{mi}; \boldsymbol{\theta}_m)\} + n\sum_{j=1}^{p_m} \rho_{\tau_m}(|\theta_{mj}|) + \boldsymbol{\nu}_m^T \boldsymbol{H}_m \boldsymbol{\theta}_m, \tag{1}$$

where $\boldsymbol{\nu}_m$ is another Lagrange multiplier imposing the constraint that $\hat{\boldsymbol{\theta}}_{m(1)} = \mathbf{0}$ (recall that $\boldsymbol{H}_m\boldsymbol{\theta}_m = \boldsymbol{\theta}_{m(1)}$). Let $\boldsymbol{\eta}_m = (\boldsymbol{\lambda}_m^T, \boldsymbol{\theta}_m^T, \boldsymbol{\nu}_m^T)^T$ be the vector of pooled parameters, and

$$\boldsymbol{Q}_{m1}(\boldsymbol{\eta}_m) = \frac{1}{n}\sum_{i=1}^{n} \frac{\boldsymbol{g}_m(\boldsymbol{\theta}_m)}{1 + \boldsymbol{\lambda}_m \boldsymbol{g}_m(\boldsymbol{\theta}_m)},$$

$$\boldsymbol{Q}_{m2}(\boldsymbol{\eta}_m) = \frac{1}{n}\sum_{i=1}^{n} \frac{\{\partial \boldsymbol{g}_m(\boldsymbol{\theta}_m)/\partial \boldsymbol{\theta}_m^T\}^T \boldsymbol{\lambda}_m}{1 + \boldsymbol{\lambda}_m \boldsymbol{g}_m(\boldsymbol{\theta}_m)} + \boldsymbol{b}_m(\boldsymbol{\theta}_m) + \boldsymbol{H}_m^T \boldsymbol{\nu}_m,$$

$$\boldsymbol{Q}_{m3}(\boldsymbol{\eta}_m) = \boldsymbol{H}_m^T \boldsymbol{\theta}_m$$

be the three components of the derivative of (1) with respect to $\boldsymbol{\eta}_m$, where

$$\boldsymbol{b}_m(\boldsymbol{\theta}_m) = (0, \ldots, 0, \rho'_{\tau_m}(|\theta_{m,q_m+1}|)\text{sign}(\theta_{m,q_m+1}), \ldots, \rho'_{\tau_m}(|\theta_{m,p_m}|)\text{sign}(\theta_{m,p_m}))^T.$$

Then the minimizer $\hat{\boldsymbol{\eta}}_m = (\hat{\boldsymbol{\lambda}}_m^T, \hat{\boldsymbol{\theta}}_m^T, \hat{\boldsymbol{\nu}}_m^T)^T$ of (1) satisfies $\boldsymbol{Q}_{mj}(\hat{\boldsymbol{\eta}}_m) = \mathbf{0}$, for $j = 1, 2, 3$. By routine derivation, we have $\|\hat{\boldsymbol{\lambda}}_m\| = O_p(n^{-1/2})$ and $\|\hat{\boldsymbol{\nu}}_m\| = O_p(n^{-1/2})$ [Qin and Lawless, 1994, Leng and Tang, 2012]. Accordingly, we expand $\boldsymbol{Q}_{mj}(\hat{\boldsymbol{\eta}}_m) = \mathbf{0}$, for $j = 1, 2, 3$, around

the true value $\boldsymbol{\eta}_m^* = (\mathbf{0}^T, \boldsymbol{\theta}_m^{*T}, \mathbf{0}^T)^T$ and have

$$\begin{pmatrix} -\boldsymbol{Q}_{m1}(\boldsymbol{\eta}_m^*) \\ \mathbf{0} \\ \mathbf{0} \end{pmatrix} = \begin{pmatrix} -\boldsymbol{A}_m & \boldsymbol{B}_m & \mathbf{0} \\ \boldsymbol{B}_m^T & \mathbf{0} & \boldsymbol{H}_m^T \\ \mathbf{0} & \boldsymbol{H}_m & \mathbf{0} \end{pmatrix} (\hat{\boldsymbol{\eta}}_m - \boldsymbol{\eta}_m^*) + o_p(n^{-1/2}). \tag{2}$$

By some algebra, we obtain the asymptotics of $\hat{\boldsymbol{\eta}}_m$ and its functions. In particular,

$$\hat{\boldsymbol{\lambda}}_m = (\boldsymbol{S}_m + \boldsymbol{P}_m)\boldsymbol{Q}_{m1}(\boldsymbol{\eta}_m^*) + o_p(n^{-1/2}),$$

where $\boldsymbol{S}_m$ and $\boldsymbol{P}_m$ are defined in Theorem 1 in the main manuscript.

Step 3. Recall that $\hat{\boldsymbol{\beta}}_{PSI}$ is the solution of

$$\sum_{i=1}^{n} \hat{p}_{mi} \boldsymbol{f}_i(\boldsymbol{\beta}) = \mathbf{0},$$

where $\hat{p}_{mi} = n^{-1}\{1 + \hat{\boldsymbol{\lambda}}_m^T \boldsymbol{g}_{mi}(\hat{\boldsymbol{\theta}}_m)\}^{-1}$. Note that $\hat{p}_{mi} = n^{-1}[1 - \hat{\boldsymbol{\lambda}}_m^T \boldsymbol{g}_{mi}\{1 + o_p(1)\}] = n^{-1}\{1 + O_p(n^{-1/2})\}$. Accordingly, the consistency of $\hat{\boldsymbol{\beta}}_{PSI}$ holds by following the proof from Van der Vaart [2000]. To derive asymptotic normality, we take expansion of $\sum_{i=1}^n \hat{p}_{mi} \boldsymbol{f}_i(\hat{\boldsymbol{\beta}}_{PSI}) = \mathbf{0}$ around $\boldsymbol{\beta}_0$:

$$\begin{aligned} \mathbf{0} &= \frac{1}{n} \sum_{i=1}^{n} [1 - \hat{\boldsymbol{\lambda}}_m^T \boldsymbol{g}_{mi}\{1 + o_p(1)\}] \boldsymbol{f}_i(\hat{\boldsymbol{\beta}}_{PSI}) \\ &= \frac{1}{n} \sum_{i=1}^{n} \boldsymbol{f}_i + \frac{1}{n} \sum_{i=1}^{n} \frac{\partial \boldsymbol{f}_i}{\partial \boldsymbol{\beta}^T} (\hat{\boldsymbol{\beta}}_{PSI} - \boldsymbol{\beta}_0) - \frac{1}{n} \sum_{i=1}^{n} \boldsymbol{f}_i \boldsymbol{g}_{mi}^T \hat{\boldsymbol{\lambda}}_m + o_p(n^{-1/2}). \end{aligned}$$

As a result, we have

$$
\begin{aligned}
n^{1/2}(\hat{\boldsymbol{\beta}}_{PSI} - \boldsymbol{\beta}_0) &= -\boldsymbol{\Gamma}^{-1}\left(n^{-1/2}\sum_{i=1}^{n}\boldsymbol{f}_i - n^{1/2}\boldsymbol{\Lambda}_m\hat{\boldsymbol{\lambda}}_m\right) + o_p(1) \\
&= -\boldsymbol{\Gamma}^{-1}\left\{n^{-1/2}\sum_{i=1}^{n}\boldsymbol{f}_i - n^{-1/2}\boldsymbol{\Lambda}_m(\boldsymbol{S}_m + \boldsymbol{P}_m)\sum_{i=1}^{n}\boldsymbol{g}_{mi}\right\} + o_p(1) \qquad (3) \\
&= -n^{-1/2}\boldsymbol{\Gamma}^{-1}\sum_{i=1}^{n}\{\boldsymbol{f}_i - \boldsymbol{\Lambda}_m(\boldsymbol{S}_m + \boldsymbol{P}_m)\boldsymbol{g}_{mi}\} + o_p(1),
\end{aligned}
$$

where $\boldsymbol{\Lambda}_m$ is defined in Theorem 1 in the main manuscript. By central limit theorem and some algebra, we have the following asymptotic variance formula as stated in Theorem 1 from the manuscript,

$$
\text{var}\left\{n^{1/2}(\hat{\boldsymbol{\beta}}_{PSI} - \boldsymbol{\beta}_0)\right\} = \boldsymbol{\Gamma}^{-1}\left(\boldsymbol{\Sigma} - \boldsymbol{\Lambda}_m\boldsymbol{S}_m\boldsymbol{\Lambda}_m^T - \boldsymbol{\Lambda}_m\boldsymbol{P}_m\boldsymbol{\Lambda}_m^T\right)\boldsymbol{\Gamma}^{-1,T}.
$$

Note that the above variance expression follows from the fact that the association matrix between $\boldsymbol{\Lambda}_m\boldsymbol{S}_m\boldsymbol{g}_{mi}$ and $\boldsymbol{\Lambda}_m\boldsymbol{P}_m\boldsymbol{g}_{mi}$ is the zero matrix.

2.3 Proof of Corollary S1

For any square matrix $\boldsymbol{A}$ with dimension d, let $\boldsymbol{A}^{(s)}$ be the s-leading principle submatrix of $\boldsymbol{A}$, for $s = 1, 2 \ldots, d$, which is the submatrix containing the first s rows and columns of $\boldsymbol{A}$. For example, for

$$
\boldsymbol{A} = \begin{pmatrix} 1 & 2 & 3 \\ 3 & 5 & 6 \\ 7 & 8 & 9 \end{pmatrix},
$$

then we have

$$
\boldsymbol{A}^{(1)} = (1),\ \boldsymbol{A}^{(2)} = \begin{pmatrix} 1 & 2 \\ 3 & 5 \end{pmatrix},\ \boldsymbol{A}^{(3)} = \boldsymbol{A}.
$$

Let $\boldsymbol{C}_m^{(s)}$ be the s-leading principle submatrix of $\boldsymbol{C}_m$ (defined in Theorem 1 in the main manuscript), for $s = 1, 2 \ldots, p_m$, then

$$\begin{aligned}\boldsymbol{P}_m &= \boldsymbol{A}_m^{-1}\boldsymbol{B}_m\boldsymbol{C}_m\boldsymbol{H}_m^T(\boldsymbol{H}_m\boldsymbol{C}_m\boldsymbol{H}_m^T)^{-1}\boldsymbol{H}_m\boldsymbol{C}_m\boldsymbol{B}_m^T\boldsymbol{A}_m^{-1} \\ &= \boldsymbol{A}_m^{-1}\boldsymbol{B}_m\boldsymbol{C}_m\begin{pmatrix}\left\{\boldsymbol{C}_m^{(q_m)}\right\}^{-1} & \\ & \boldsymbol{0}\end{pmatrix}\boldsymbol{C}_m\boldsymbol{B}_m^T\boldsymbol{A}_m^{-1}.\end{aligned}$$

Without loss of generality, suppose that the penalty term of the last entry of $\boldsymbol{\theta}_{m(1)}$, that is $\rho_m(\theta_{m,q_m})$, is dropped. Following a similar argument as in the proof of Theorem 1, we know the corresponding estimator of $\hat{\boldsymbol{\beta}}_{PSI}^{(q_m-1)}$ is consistent, with asymptotic variance $\text{var}\left\{n^{1/2}(\hat{\boldsymbol{\beta}}_{PSI}^{(q_m-1)} - \boldsymbol{\beta}_0)\right\} = \boldsymbol{\Gamma}^{-1}\left(\boldsymbol{\Sigma} - \boldsymbol{\Lambda}_m\boldsymbol{S}_m\boldsymbol{\Lambda}_m^T - \boldsymbol{\Lambda}_m\boldsymbol{P}_m^{(q_m-1)}\boldsymbol{\Lambda}_m^T\right)\boldsymbol{\Gamma}^{-1,T}$, where

$$\boldsymbol{P}_m^{(q_m-1)} = \boldsymbol{A}_m^{-1}\boldsymbol{B}_m\boldsymbol{C}_m\begin{pmatrix}\left\{\boldsymbol{C}_m^{(q_m-1)}\right\}^{-1} & \\ & \boldsymbol{0}\end{pmatrix}\boldsymbol{C}_m\boldsymbol{B}_m^T\boldsymbol{A}_m^{-1}.$$

Because $\boldsymbol{C}_m$ is in general positive-definite, $\boldsymbol{C}_m^{(q_m-1)}$ and $\boldsymbol{C}_m^{(q_m)}$ are also positive-definite. Note that $\boldsymbol{C}_m^{(q_m-1)}$ is also the $(q_m - 1)$-leading principle submatrix of $\boldsymbol{C}_m^{(q_m)}$. As a result, we have

$$\left\{\boldsymbol{C}_m^{(q_m)}\right\}^{-1} \geqslant \begin{pmatrix}\left\{\boldsymbol{C}_m^{(q_m-1)}\right\}^{-1} & \\ & 0\end{pmatrix},$$

where $\boldsymbol{\Psi}_1 \geqslant \boldsymbol{\Psi}_2$ means $\boldsymbol{\Psi}_1 - \boldsymbol{\Psi}_2$ is nonnegative-definite for any matrices $\boldsymbol{\Psi}_1$ and $\boldsymbol{\Psi}_2$. Therefore, it follows immediately that $\text{var}\left\{n^{1/2}(\hat{\boldsymbol{\beta}}_{PSI}^{(q_m-1)} - \boldsymbol{\beta}_0)\right\} \geqslant \text{var}\left\{n^{1/2}(\hat{\boldsymbol{\beta}}_{PSI} - \boldsymbol{\beta}_0)\right\}$, and we prove that dropping a penalty term for an entry of $\boldsymbol{\theta}_{m(1)}$ will result in a less efficient estimator of $\boldsymbol{\beta}$. The above proof strategy can be easily generalized if multiple penalty terms are dropped, thus omitted here.

2.4 Proof of Corollary S2

Recall that $\boldsymbol{R}_m = \boldsymbol{A}_m(\boldsymbol{S}_m + \boldsymbol{P}_m)$. By (2) in the proof of Theorem 1, we have

$$\frac{1}{n}\sum_{i=1}^{n} \boldsymbol{g}_{mi}(\hat{\boldsymbol{\theta}}_m) = \boldsymbol{R}_m \boldsymbol{Q}_{m1}(\boldsymbol{\eta}_m^*) + o_p(n^{-1/2}) = O_p(n^{-1/2}).$$

Therefore, for any estimating equation $n^{-1}\sum_{i=1}^{n}\left\{\boldsymbol{f}_i(\boldsymbol{\beta}) + \boldsymbol{F}_m \boldsymbol{g}_{mi}(\hat{\boldsymbol{\theta}}_m)\right\} = o_p(n^{-1/2})$, we have that the solution of the estimating equation $\hat{\boldsymbol{\beta}}$ is consistent to the true value $\boldsymbol{\beta}_0$ by Van der Vaart [2000]. Taking expansion around $\boldsymbol{\beta}_0$, we have

$$\boldsymbol{0} = \frac{1}{n}\sum_{i=1}^{n} \boldsymbol{f}_i + \frac{1}{n}\sum_{i=1}^{n} \frac{\partial \boldsymbol{f}_i}{\partial \boldsymbol{\beta}^T}(\hat{\boldsymbol{\beta}} - \boldsymbol{\beta}_0) - \frac{1}{n}\boldsymbol{F}_m \sum_{i=1}^{n} \boldsymbol{g}_{mi}(\hat{\boldsymbol{\theta}}_m) + o_p(n^{-1/2}).$$

As a result, we have

$$\begin{aligned} n^{1/2}(\hat{\boldsymbol{\beta}} - \boldsymbol{\beta}_0) &= -\boldsymbol{\Gamma}^{-1}\left\{n^{-1/2}\sum_{i=1}^{n} \boldsymbol{f}_i - n^{-1/2}\boldsymbol{F}_m \boldsymbol{R}_m \boldsymbol{Q}_{m1}(\boldsymbol{\eta}_m^*)\right\} + o_p(1) \\ &= -n^{-1/2}\boldsymbol{\Gamma}^{-1}\sum_{i=1}^{n}(\boldsymbol{f}_i - \boldsymbol{F}_m \boldsymbol{R}_m \boldsymbol{g}_{mi}) + o_p(1). \end{aligned}$$

By the projection theory [Bickel et al., 1993, Tsiatis, 2006], in order to minimize var $\left\{n^{1/2}(\hat{\boldsymbol{\beta}} - \boldsymbol{\beta}_0)\right\}$, we need to find a $\boldsymbol{F}_m^*$ such that $\boldsymbol{F}_m^* \boldsymbol{R}_m \boldsymbol{g}_m$ is the projection of $\boldsymbol{f}$ onto the subspace spanned by $\boldsymbol{R}_m \boldsymbol{g}_m$. Equivalently, $\boldsymbol{F}_m^*$ is a solution of the following equation

$$E\left\{\boldsymbol{f}(\boldsymbol{R}_m \boldsymbol{g}_m)^T\right\} = \boldsymbol{F}_m^* E\left\{\boldsymbol{R}_m \boldsymbol{g}_m (\boldsymbol{R}_m \boldsymbol{g}_m)^T\right\}.$$

Recall that $\boldsymbol{\Lambda}_m = E(\boldsymbol{f}\boldsymbol{g}_m^T)$, $\boldsymbol{A}_m = E(\boldsymbol{g}_m\boldsymbol{g}_m^T)$, and $\boldsymbol{R}_m$ is symmetric, the equation above can be rewritten as

$$\boldsymbol{\Lambda}_m(\boldsymbol{S}_m + \boldsymbol{P}_m)\boldsymbol{A}_m = \boldsymbol{F}_m^*\boldsymbol{A}_m(\boldsymbol{S}_m + \boldsymbol{P}_m)\boldsymbol{A}_m(\boldsymbol{S}_m + \boldsymbol{P}_m)\boldsymbol{A}_m.$$

Some calculations show that $(\boldsymbol{S}_m + \boldsymbol{P}_m)\boldsymbol{A}_m(\boldsymbol{S}_m + \boldsymbol{P}_m) = \boldsymbol{S}_m + \boldsymbol{P}_m$ [Qin and Lawless, 1995], so the equation above can be rewritten as

$$\boldsymbol{\Lambda}_m(\boldsymbol{S}_m + \boldsymbol{P}_m)\boldsymbol{A}_m = \boldsymbol{F}_m^*\boldsymbol{A}_m(\boldsymbol{S}_m + \boldsymbol{P}_m)\boldsymbol{A}_m.$$

Note that $\boldsymbol{F}_m^* = \boldsymbol{\Lambda}_m\boldsymbol{A}_m^{-1}$ is one solution, which means any estimator with influence function $-\boldsymbol{\Gamma}^{-1}\{\boldsymbol{f}_i - \boldsymbol{\Lambda}_m(\boldsymbol{S}_m + \boldsymbol{P}_m)\boldsymbol{g}_{mi}\}$ is the optimal estimator. Examining (3) in the proof of Theorem 1, we conclude that $\hat{\boldsymbol{\beta}}_{PSI}$ is optimal.

2.5 Proof of Theorem S1

The logic of this proof is similar to that of Theorem 1, and therefore we provide only a sketch. Recall that $\hat{\boldsymbol{\beta}}_{avg}$ is the solution of

$$\sum_{i=1}^{n}\left(\sum_{m=1}^{M} w_m\hat{p}_{mi}\right)\boldsymbol{f}_i(\boldsymbol{\beta}) = \boldsymbol{0},$$

where $\hat{p}_{mi} = n^{-1}\{1 + \hat{\boldsymbol{\lambda}}_m^T\boldsymbol{g}_{mi}(\hat{\boldsymbol{\theta}}_m)\}^{-1}$. Note that $\hat{p}_{mi} = n^{-1}[1 - \hat{\boldsymbol{\lambda}}_m^T\boldsymbol{g}_{mi}\{1 + o_p(1)\}] = n^{-1}\{1 + O_p(n^{-1/2})\}$. Accordingly, the consistency of $\hat{\boldsymbol{\beta}}_{avg}$ holds by following the proof from

Van der Vaart [2000]. To derive asymptotic normality, we take expansion around $\boldsymbol{\beta}_0$:

$$\begin{aligned}
\mathbf{0} &= \frac{1}{n}\sum_{i=1}^{n}\left[1-\sum_{m=1}^{M} w_m\hat{\boldsymbol{\lambda}}_m^T\boldsymbol{g}_{mi}\{1+o_p(1)\}\right]\boldsymbol{f}_i(\hat{\boldsymbol{\beta}}_{avg}) \\
&= \frac{1}{n}\sum_{i=1}^{n}\boldsymbol{f}_i+\frac{1}{n}\sum_{i=1}^{n}\frac{\partial \boldsymbol{f}_i}{\partial \boldsymbol{\beta}^T}(\hat{\boldsymbol{\beta}}_{avg}-\boldsymbol{\beta}_0)-\frac{1}{n}\sum_{i=1}^{n}\sum_{m=1}^{M} w_m\boldsymbol{f}_i\boldsymbol{g}_{mi}^T\hat{\boldsymbol{\lambda}}_m+o_p(n^{-1/2}).
\end{aligned}$$

As a result, we have

$$\begin{aligned}
n^{1/2}(\hat{\boldsymbol{\beta}}_{avg}-\boldsymbol{\beta}_0) &= -\boldsymbol{\Gamma}^{-1}\left(n^{-1/2}\sum_{i=1}^{n}\boldsymbol{f}_i-n^{1/2}\sum_{m=1}^{M} w_m\boldsymbol{\Lambda}_m\hat{\boldsymbol{\lambda}}_m\right)+o_p(1) \\
&= -\boldsymbol{\Gamma}^{-1}\left\{n^{-1/2}\sum_{i=1}^{n}\boldsymbol{f}_i-n^{-1/2}\sum_{m=1}^{M} w_m\boldsymbol{\Lambda}_m(\boldsymbol{S}_m+\boldsymbol{P}_m)\sum_{i=1}^{n}\boldsymbol{g}_{mi}\right\}+o_p(1) \qquad (4) \\
&= -n^{-1/2}\boldsymbol{\Gamma}^{-1}\sum_{i=1}^{n}\left\{\boldsymbol{f}_i-\sum_{m=1}^{M} w_m\boldsymbol{\Lambda}_m(\boldsymbol{S}_m+\boldsymbol{P}_m)\boldsymbol{g}_{mi}\right\}+o_p(1),
\end{aligned}$$

where $\boldsymbol{\Lambda}_m$ is defined in Theorem 1 in the main manuscript. By the central limit theorem and some algebra, we have the following asymptotic variance formula, as stated in Theorem S1:

$$\boldsymbol{\Gamma}^{-1}\left\{\boldsymbol{\Sigma}-\sum_{m=1}^{M} w_m^2\boldsymbol{\Lambda}_m(\boldsymbol{S}_m+\boldsymbol{P}_m)\boldsymbol{\Lambda}_m^T+\sum_{i\neq j}^{M} w_iw_j\boldsymbol{\Lambda}_i(\boldsymbol{S}_i+\boldsymbol{P}_i)\tilde{\boldsymbol{A}}_{ij}(\boldsymbol{S}_j+\boldsymbol{P}_j)\boldsymbol{\Lambda}_j^T\right\}\boldsymbol{\Gamma}^{-1,T}.$$

2.6 Proof of Theorem 2

Some preliminaries that can be easily obtained from the previous derivations are provided here: first, by the proof of Theorem 1, $\{\hat{\boldsymbol{\theta}}_m\}_{m=1}^M$ are consistent estimators for $\{\boldsymbol{\theta}_m^*\}_{m=1}^M$, so the pooled estimator $\hat{\boldsymbol{\Theta}}$ is consistent for $\boldsymbol{\Theta}^*$. Second, let $\boldsymbol{\Theta}_{(1)}^*$ be the zero component of $\boldsymbol{\Theta}^*$. The conclusion in Theorem 1 implies that $P(\hat{\boldsymbol{\Theta}}_{(1)}^*=\mathbf{0})\to 1$. Also, by (2) in the proof

of Theorem 1, we have

$$\frac{1}{n}\sum_{i=1}^{n}\boldsymbol{G}_i(\hat{\boldsymbol{\Theta}}) = \frac{1}{n}\boldsymbol{R}\sum_{i=1}^{n}\boldsymbol{G}_i + o_p(n^{-1/2}),$$

where $\boldsymbol{R}$ is defined in Section 3.2 in the main manuscript. Note that the sample variance of $\hat{\boldsymbol{R}}\boldsymbol{G}(\tilde{\boldsymbol{D}}_i;\hat{\boldsymbol{\Theta}})$, $i = 1, 2, \ldots, n$, is $\boldsymbol{W} + o_p(1)$, where $\boldsymbol{W}$ is defined in Theorem 2 in the main manuscript. Thus, $\hat{K}$, $\hat{\boldsymbol{\Phi}}$ and $\hat{\boldsymbol{U}}$ (defined in Section 3.2 in the main manuscript) converge to K, $\boldsymbol{\Phi}$ and $\boldsymbol{U}$ (defined in Theorem 2 in the main manuscript) in probability, respectively [Anderson, 1963, Shen et al., 2016]. As a result, we have $\hat{p}_i = n^{-1}\{1 + O_p(n^{-1/2})\}$, and the consistency of $\hat{\boldsymbol{\beta}}_{PEPSI}$ follows Van der Vaart [2000].

Now we derive the property of asymptotic normality. First, taking Taylor expansion of $\sum_{i=1}^{n}\hat{p}_i\boldsymbol{f}_i(\hat{\boldsymbol{\beta}}_{PEPSI}) = \boldsymbol{0}$ around $\boldsymbol{\beta}_0$ and using the property that $\boldsymbol{R}$ is idempotent, we have

$$\begin{aligned}\boldsymbol{0} &= \frac{1}{n}\sum_{i=1}^{n}\left\{1 - \frac{1}{n}\boldsymbol{G}_i^T(\hat{\boldsymbol{\Theta}})\hat{\boldsymbol{R}}^T\hat{\boldsymbol{U}}\hat{\boldsymbol{\Phi}}^{-1}\hat{\boldsymbol{U}}^T\hat{\boldsymbol{R}}\sum_{i=1}^{n}\boldsymbol{G}_i(\hat{\boldsymbol{\Theta}})\right\}\boldsymbol{f}_i(\hat{\boldsymbol{\beta}}_{PEPSI}) \\ &= \frac{1}{n}\sum_{i=1}^{n}\boldsymbol{f}_i + \boldsymbol{\Gamma}(\hat{\boldsymbol{\beta}}_{PEPSI} - \boldsymbol{\beta}_0) - \frac{1}{n}\boldsymbol{\Lambda}\boldsymbol{R}^T\boldsymbol{U}\boldsymbol{\Phi}^{-1}\boldsymbol{U}^T\boldsymbol{R}\sum_{i=1}^{n}\boldsymbol{G}_i + o_p(n^{-1/2}).\end{aligned}$$

As a result, we have

$$n^{1/2}(\hat{\boldsymbol{\beta}}_{PEPSI} - \boldsymbol{\beta}_0) = -n^{-1/2}\boldsymbol{\Gamma}^{-1}\sum_{i=1}^{n}\left(\boldsymbol{f}_i - \boldsymbol{\Lambda}\boldsymbol{R}^T\boldsymbol{U}\boldsymbol{\Phi}^{-1}\boldsymbol{U}^T\boldsymbol{R}\boldsymbol{G}_i\right) + o_p(1). \quad (5)$$

By central limit theorem and some algebra, the asymptotic normality holds with the following asymptotic variance formula

$$\operatorname{var}\left\{n^{1/2}(\hat{\boldsymbol{\beta}}_{PEPSI} - \boldsymbol{\beta}_0)\right\} = \boldsymbol{\Gamma}^{-1}\left(\boldsymbol{\Sigma} - \boldsymbol{\Lambda}\boldsymbol{R}^T\boldsymbol{U}\boldsymbol{\Phi}^{-1}\boldsymbol{U}^T\boldsymbol{R}\boldsymbol{\Lambda}^T\right)\boldsymbol{\Gamma}^{-1,T}.$$

2.7 Proof of Corollary 1

The proof is similar to the one of Corollary S2. By (2) in the proof of Theorem 1, we know that, for $1 \leqslant m \leqslant M$,

$$\frac{1}{n}\sum_{i=1}^{n} \boldsymbol{g}_{mi}(\hat{\boldsymbol{\theta}}_m) = \boldsymbol{R}_m \boldsymbol{Q}_{m1}(\boldsymbol{\eta}_m^*) + o_p(n^{-1/2}) = O_p(n^{-1/2}).$$

Therefore, for any estimating equation $n^{-1}\sum_{i=1}^{n}\left\{\boldsymbol{f}(\boldsymbol{\beta}) + \sum_{m=1}^{M} \boldsymbol{F}_m \boldsymbol{g}_{mi}(\hat{\boldsymbol{\theta}}_m)\right\} = o_p(n^{-1/2})$, we know the solution of the estimating equation $\hat{\boldsymbol{\beta}}$ is consistent to $\boldsymbol{\beta}_0$ by Van der Vaart [2000]. Taking expansion around $\boldsymbol{\beta}_0$, we have

$$\mathbf{0} = \frac{1}{n}\sum_{i=1}^{n} \boldsymbol{f}_i + \frac{1}{n}\sum_{i=1}^{n} \frac{\partial \boldsymbol{f}_i}{\partial \boldsymbol{\beta}^T}(\hat{\boldsymbol{\beta}} - \boldsymbol{\beta}_0) - \frac{1}{n}\sum_{i=1}^{n}\sum_{m=1}^{M} \boldsymbol{F}_m \boldsymbol{g}_{mi}(\hat{\boldsymbol{\theta}}_m) + o_p(n^{-1/2}).$$

As a result, we have

$$\begin{aligned} n^{1/2}(\hat{\boldsymbol{\beta}} - \boldsymbol{\beta}_0) &= -n^{-1/2}\boldsymbol{\Gamma}^{-1}\sum_{i=1}^{n}\left(\boldsymbol{f}_i - \sum_{m=1}^{M} \boldsymbol{F}_m \boldsymbol{R}_m \boldsymbol{g}_{mi}\right) + o_p(1) \\ &= -n^{-1/2}\boldsymbol{\Gamma}^{-1}\sum_{i=1}^{n}(\boldsymbol{f}_i - \boldsymbol{F}\boldsymbol{R}\boldsymbol{G}_i) + o_p(1), \end{aligned}$$

where $\boldsymbol{F} = (\boldsymbol{F}_1, \ldots, \boldsymbol{F}_m)$. By projection theory [Bickel et al., 1993, Tsiatis, 2006], in order to minimize var$\left\{n^{1/2}(\hat{\boldsymbol{\beta}} - \boldsymbol{\beta}_0)\right\}$, we need to find a $\boldsymbol{F}^*$ such that $\boldsymbol{F}^*\boldsymbol{R}\boldsymbol{G}$ is the projection of $\boldsymbol{f}$ onto the subspace spanned by $\boldsymbol{R}\boldsymbol{G}$. Equivalently, $\boldsymbol{F}^*$ is a solution of the following equation

$$E\left\{\boldsymbol{f}(\boldsymbol{R}\boldsymbol{G})^T\right\} = \boldsymbol{F}^* E\left\{\boldsymbol{R}\boldsymbol{G}(\boldsymbol{R}\boldsymbol{G})^T\right\}.$$

It can be rewritten as

$$\boldsymbol{\Lambda}\boldsymbol{R}^T = \boldsymbol{F}^*\boldsymbol{W}.$$

Note that $\boldsymbol{F}^* = \boldsymbol{\Lambda}\boldsymbol{R}^T\boldsymbol{W}^- = \boldsymbol{\Lambda}\boldsymbol{R}^T\boldsymbol{U}\boldsymbol{\Phi}^{-1}\boldsymbol{U}^T$ is a solution, where $\boldsymbol{\Psi}^-$ means the Moore–Penrose inverse of any matrix $\boldsymbol{\Psi}$. Hence, any estimator with influence function $-\boldsymbol{\Gamma}^{-1}(\boldsymbol{f}_i - \boldsymbol{\Lambda}\boldsymbol{R}^T\boldsymbol{U}\boldsymbol{\Phi}^{-1}\boldsymbol{U}^T\boldsymbol{R}\boldsymbol{G}_i)$ is the optimal estimator. Examining (5) in the proof of Theorem 2, we know that $\hat{\boldsymbol{\beta}}_{PEPSI}$ is optimal.

3 More simulation details and results

In this section, we present more details and results regarding numerical simulations. First, the coefficient matrix in Case 2 in the manuscript is

$$\boldsymbol{\Theta} = \begin{pmatrix} 1 & 0 & 0 & 0 & 1 & 1 & 1 & 1 & 0 & 0 & 1 & 1 & \dots & 1 \\ 1 & 0 & 0 & 0 & 1 & 1 & 0 & 0 & 1 & 1 & 1 & 1 & \dots & 1 \\ 0 & 1 & 1 & 1 & 0 & 0 & 0 & 0 & 1 & 1 & 1 & 1 & \dots & 1 \\ 0 & 1 & 1 & 1 & 0 & 0 & 1 & 1 & 0 & 0 & 1 & 1 & \dots & 1 \end{pmatrix}.$$

Next, we investigate more scenarios as follow.

3.1 Naive estimator and VIS estimators with/without oracle information

We examined the performance of $\hat{\boldsymbol{\beta}}_{naive}$ and $\hat{\boldsymbol{\beta}}_{VIS}$ with/without oracle information. We adopted the setting as in Case 1 in the main manuscript, where the sample size was $n = 600$, the correlation was $\rho = 0.8$, and there were two zeros in the working model coefficients (θ_{13} and θ_{14}). Specifically, we considered 3 scenarios for $\hat{\boldsymbol{\beta}}_{VIS}$: (1) no oracle information, i.e., no prior knowledge to inform which working model parameters are zero; (2) partial oracle information, i.e., only a subset of zero-valued parameters was known a priori (θ_{13}); (3) full

oracle information, i.e., all zero entries were known a priori (θ_{13} and θ_{14}).

The results are summarized in Table S1. When there was no oracle information, $\hat{\boldsymbol{\beta}}_{VIS}$ reduced to $\hat{\boldsymbol{\beta}}_{naive}$. As we knew more information about which parameters were zero a priori, the resulting estimator became more efficient. $\hat{\boldsymbol{\beta}}_{VIS}$ with full oracle information was similar to $\hat{\boldsymbol{\beta}}_{PSI}$. In general, however, $\hat{\boldsymbol{\beta}}_{PSI}$ is data-driven and does not require prior knowledge about the working models, which is typical the case in real-world data application. Therefore, $\hat{\boldsymbol{\beta}}_{PSI}$ is more preferred in practice.

Table S1: Simulation results of the naive and VIS estimators with and without prior knowledge about the zero coefficients. All results, except RE, are multiplied by 100.

	Bias	MCSD	SE	CP	RE	Bias	MCSD	SE	CP	RE
	Naive					VIS no oracle				
β_0	0.0	4.1	4.1	95	1.0	0.0	4.1	4.1	95	1.0
β_1	0.0	4.7	4.7	95	1.0	0.0	4.7	4.7	95	1.0
β_2	0.0	5.4	5.3	95	1.0	0.0	5.4	5.3	95	1.0
β_3	-0.1	5.3	5.3	94	1.0	-0.1	5.3	5.3	94	1.0
β_4	0.0	4.7	4.7	95	1.0	0.0	4.7	4.7	95	1.0
	VIS partial oracle					VIS full oracle				
β_0	0.0	4.1	4.1	95	1.0	0.0	4.1	4.1	95	1.0
β_1	0.0	4.7	4.7	95	1.0	0.0	4.8	4.7	95	1.0
β_2	0.0	5.0	5.0	95	1.1	-0.1	5.0	4.9	94	1.2
β_3	0.0	3.2	3.2	95	2.8	0.0	3.2	3.2	95	2.8
β_4	0.0	4.4	4.3	95	1.2	0.1	2.8	2.8	95	2.7

Abbreviations: MCSD, Monte Carlo standard deviation; SE, estimated standard error; RE, relative efficiency over the naive estimator; CP, 95% coverage probability.

3.2 Equivalence of the PSI and PEPSI estimators

We considered a case to compare $\hat{\boldsymbol{\beta}}_{PSI}$ and $\hat{\boldsymbol{\beta}}_{PEPSI}$ when integrating a single secondary outcome. We adopted the same data generating process as in Case 1. We considered a scenario where $n = 300$ and $\rho = 0.8$.

The results are summarized in Table S2. We can infer that the simulation results of $\hat{\boldsymbol{\beta}}_{PSI}$ and $\hat{\boldsymbol{\beta}}_{PEPSI}$ were basically the same, confirming that they are equivalent when integrating

Table S2: Simulation results for comparing the PSI and PEPSI estimators. All results, except RE, are multiplied by 100.

	PSI					PEPSI				
	Bias	MCSD	SE	CP	RE	Bias	MCSD	SE	CP	RE
β_0	0.0	5.9	5.7	94	1.0	0.0	5.9	5.7	94	1.0
β_1	0.0	6.7	6.6	95	1.0	0.0	6.7	6.6	95	1.0
β_2	0.1	7.2	6.9	94	1.1	0.1	7.2	6.9	94	1.1
β_3	-0.1	4.9	4.5	94	2.4	-0.1	4.9	4.5	94	2.3
β_4	0.1	4.4	4.1	94	2.3	0.1	4.4	4.1	94	2.3

Abbreviations: MCSD, Monte Carlo standard deviation; SE, estimated standard error; RE, relative efficiency over the naive estimator; CP, 95% coverage probability.

one secondary outcome.

3.3 One binary primary outcome and multiple continuous secondary outcomes

We evaluated a case where the primary outcome was binary. We adopted the same data generating process for the covariate $\boldsymbol{X}$ and the secondary outcomes $\{\tilde{Y}_1, \ldots, \tilde{Y}_{50}\}$ as in Case 2, except that the entries of the correlation matrix for $\{\tilde{Y}_1, \ldots, \tilde{Y}_{50}\}$ became constant and equaled to 0.6. The mean of the primary outcome was $E(Y = 1|\boldsymbol{X}) = \{1 + \exp(-\beta_0 - \boldsymbol{\beta}^T \boldsymbol{X})\}^{-1}$ with $\beta_0 = 0$ and $\boldsymbol{\beta} = (0.1, 0.1, 0, 0)^T$. To generate associated primary and secondary outcomes conditional on the covariates, we first generated a gaussian-distributed random variable $\tilde{Y}_0$ with mean 0 and variance 1, which was correlated with $\{\tilde{Y}_1, \ldots, \tilde{Y}_{50}\}$. Then we set $Y = 1$ when $\tilde{Y}_0$ exceeded the $1 - E(Y = 1|\boldsymbol{X})$ percentile of the standard normal distribution and set $Y = 0$ otherwise. Following the above generation, the resulting correlations between Y and $\{\tilde{Y}_1, \ldots, \tilde{Y}_{50}\}$ were about $0.68, 0.68, 0.48, 0.48, \ldots, 0.48$, respectively.

The results are summarized in Table S3, where overall patterns and conclusions were found to be consistent with our previous discussion in Case 2, indicating that our proposed

Table S3: Simulation results of the PEPSI and Average scheme estimators under a binary primary outcome and multiple continuous secondary outcomes. All results, except RE, are multiplied by 100.

			PEPSI					Averaging scheme				
			Bias	MCSD	SE	CP	RE	Bias	MCSD	SE	CP	RE
n=300	Integrating 10 outcomes	β_0	0.1	12.1	11.6	94	1.0	0.1	11.9	11.7	95	1.0
		β_1	0.1	11.9	11.3	94	1.3	0.1	13.0	12.9	95	1.1
		β_2	0.2	13.1	12.3	94	1.4	0.1	14.2	13.8	94	1.2
		β_3	-0.3	11.7	10.9	93	1.7	-0.3	11.8	11.4	94	1.7
		β_4	0.1	10.6	9.8	93	1.7	0.0	10.7	10.2	95	1.7
	Integrating 50 outcomes	β_0	0.1	12.1	11.4	94	0.9	0.1	11.6	11.7	95	1.0
		β_1	0.1	10.7	9.6	92	1.7	0.1	11.4	11.1	94	1.5
		β_2	0.2	11.9	10.5	92	1.7	0.1	12.3	12.0	95	1.6
		β_3	-0.2	11.7	10.4	92	1.7	-0.2	12.2	11.9	94	1.6
		β_4	0.0	10.7	9.5	92	1.7	0.0	11.4	11.0	94	1.5
n=600	Integrating 10 outcomes	β_0	0.0	8.3	8.2	95	1.0	0.0	8.2	8.2	95	1.0
		β_1	0.1	8.2	8.0	95	1.4	0.1	9.1	9.1	95	1.1
		β_2	0.3	9.0	8.7	94	1.4	0.2	9.9	9.7	95	1.2
		β_3	-0.1	8.0	7.7	94	1.8	-0.1	8.2	8.0	94	1.7
		β_4	0.0	7.1	6.9	94	1.8	0.0	7.3	7.2	94	1.7
	Integrating 50 outcomes	β_0	0.0	8.4	8.1	94	1.0	-0.1	8.3	8.2	95	1.0
		β_1	0.2	7.3	6.8	93	1.8	0.2	7.9	7.8	95	1.5
		β_2	0.0	7.9	7.5	94	1.9	0.0	8.6	8.4	95	1.6
		β_3	0.0	7.8	7.5	94	1.9	0.0	8.4	8.4	95	1.6
		β_4	0.0	7.1	6.8	94	1.8	0.1	7.8	7.7	95	1.5

Abbreviations: MCSD, Monte Carlo standard deviation; SE, estimated standard error; RE, relative efficiency over the naive estimator; CP, 95% coverage probability.

method also worked well and outperformed the existing scheme with a binary primary outcome.

3.4 Low primary and secondary outcomes correlations

We considered a case where the correlations between the primary and secondary outcomes are low. We adopted the same data generating process as described in Case 2 in the main manuscript, except that the conditional correlation between Y and $(\tilde{Y}_1, \ldots, \tilde{Y}_{50})$ given $\boldsymbol{X}$ was $\rho = 0.2$ or $\rho = 0$, with a sample size $n = 600$. We considered a case to integrate all 50

secondary outcomes. Again, we evaluated and compared $\hat{\boldsymbol{\beta}}_{PEPSI}$ and $\hat{\boldsymbol{\beta}}_{avg}$.

Table S4: Simulation results of the low correlation case. All results, except RE, are multiplied by 100.

		PEPSI					Averaging scheme				
		Bias	MCSD	SE	CP	RE	Bias	MCSD	SE	CP	RE
$\rho = 0.2$	β_0	-0.1	4.1	4.0	94	1.0	0.0	4.1	4.1	95	1.0
	β_1	0.0	4.6	4.4	94	1.0	0.0	4.6	4.6	95	1.0
	β_2	0.0	5.2	5.0	94	1.0	0.0	5.2	5.1	95	1.0
	β_3	0.1	5.3	5.0	93	1.0	0.1	5.2	5.1	95	1.0
	β_4	-0.1	4.7	4.4	94	1.0	-0.1	4.6	4.6	95	1.0
$\rho = 0$	β_0	-0.1	4.1	4.0	94	1.0	-0.1	4.1	4.1	95	1.0
	β_1	0.0	4.8	4.6	93	0.9	0.0	4.7	4.7	95	1.0
	β_2	0.0	5.4	5.1	94	1.0	0.0	5.3	5.2	95	1.0
	β_3	0.1	5.4	5.1	94	0.9	0.1	5.3	5.2	95	1.0
	β_4	-0.1	4.9	4.6	94	0.9	-0.1	4.7	4.7	94	1.0

Abbreviations: MCSD, Monte Carlo standard deviation; SE, estimated standard error; RE, relative efficiency over the naive estimator; CP, 95% coverage probability.

The results are summarized in Table S4. We found that the proposed estimators remained consistent, with CP close to the nominal level (95%) under settings with low correlations. The relative efficiency was close to 1 in most cases, and was slightly below than 1 when $\rho = 0$, which was expected because incorporating secondary outcomes not associated with the primary outcome introduced pure noise into the model. Therefore, it is recommended to integrate secondary outcomes that are moderately or highly associated with the primary outcome (e.g., with an absolute correlation coefficient greater than 0.4).

3.5 Low signal-to-noise ratio (SNR)

We considered a case with a lower SNR and adopted the same data generating process as in Case 2 in the main manuscript, except that all primary and secondary coefficient parameters were halved. As a result, the SNR was lowered. We considered a scenario where the sample size was $n = 600$, and we integrated all 50 secondary outcomes.

Table S5: Simulation results of the low SNR case. All results, except RE, are multiplied by 100.

	PEPSI					Averaging scheme				
	Bias	MCSD	SE	CP	RE	Bias	MCSD	SE	CP	RE
β_0	0.0	4.2	4.0	94	1.0	0.0	4.1	4.1	95	1.0
β_1	0.0	2.9	2.7	93	2.7	0.0	3.5	3.4	94	1.8
β_2	0.0	3.1	2.8	93	3.1	0.0	3.8	3.6	94	2.0
β_3	0.0	3.1	2.8	93	3.1	0.0	3.8	3.6	94	2.0
β_4	0.1	2.9	2.7	93	2.7	0.1	3.6	3.4	94	1.8

Abbreviations: MCSD, Monte Carlo standard deviation; SE, estimated standard error; RE, relative efficiency over the naive estimator; CP, 95% coverage probability.

The results are summarized in Table S5. We found that the proposed estimators remained consistent, with CP close to the nominal level (95%). The relative efficiency was basically the same as the one with high SNR (Table 2 of the manuscript), suggesting the robustness of our proposal, even under the setting with a lower SNR.

3.6 Predicted primary outcome

We mimicked the analysis procedure in the application section in the manuscript. We adopted the data generating model considered in Case 2 in the manuscript. We first generated a training dataset of size $1,000$ and fitted a XGBoost model predicting the primary outcome Y based on the covariates $\boldsymbol{X}$ and secondary outcomes $\tilde{\boldsymbol{Y}}$. Fixing the predicted model, we ran $1,000$ times Monte Carlo replications. In each replication, we generated an independent validation dataset of size $1,000$, and predicted the primary outcome $\hat{Y}$ based on the predicted model using the covariates and secondary outcomes in the validation set. Then, we treated $\hat{Y}$ as the primary outcome and the remaining procedure was the same as in Case 2 in the manuscript. Note that the limit values of the parameter estimates of interest depend on the specified model. Therefore, we generated an additional large, independent validation dataset of size 1×10^7 to numerically determine the limit values as

a benchmark.

Table S6: Simulation results of the primary outcome predicted by the covariates and secondary outcomes. All results except RE are multiplied by 100.

Parameter	Limit	Bias	MCSD	SE	CP	RE
β_0	101	-0.1	3.0	2.9	93	1.0
β_1	99	0.0	1.8	1.7	94	3.7
β_2	102	0.0	1.9	1.7	94	4.4
β_3	96	0.0	1.7	1.7	95	4.7
β_4	101	0.0	1.6	1.6	94	4.1

Abbreviations: Limit, limit values of parameter estimates based on the trained model; MCSD, Monte Carlo standard deviation; SE, estimated standard error; RE, relative efficiency over the naive estimator; CP, 95% coverage probability.

The results are summarized in Table S6. The limit value was close to the true value $\boldsymbol{\beta}_0 = (1,1,1,1,1)^T$, indicating that the fitted XGBoost model provided an good prediction to the primary outcome. Besides, the bias, MCSD, SE and CP showed that our estimator was consistent and our asymptotic theory worked well. Comparing Table 2 in the manuscript and Table S6, we found that the efficiency gain (RE) of $\hat{\boldsymbol{\beta}}_{PEPSI}$ was even much higher when using the predicted primary outcome than using the raw primary outcome. This was because the predicted primary outcome had higher association with the secondary outcomes.

3.7 Small sample sizes

We examined the performance of $\hat{\boldsymbol{\beta}}_{PEPSI}$ and $\hat{\boldsymbol{\beta}}_{avg}$ under small sample sizes. We adopted the data generation process from Case 2 in the main manuscript, except that the sample sizes were set to $n = 80$ and $n = 150$. The results are summarized in Table S7. We found that when small sample sizes were small, both estimators remained consistent. Moreover, these two estimators were more efficient than the naive estimator, except for $\hat{\boldsymbol{\beta}}_{PEPSI}$ when integrating 50 secondary outcomes with $n = 80$. Besides, the asymptotic variances tended

to underestimate the uncertainty of the estimators and, therefore, the CP was below the nominal levels for both estimators. However, $\hat{\boldsymbol{\beta}}_{avg}$ achieved slightly better CP, which was closer to the nominal level (95%) when sample sizes were small.

Table S7: Simulation results of the PEPSI and Averaging scheme estimators under small sample sizes. All result, except RE, are multiplied by 100.

			PEPSI					Averaging scheme				
			Bias	MCSD	SE	CP	RE	Bias	MCSD	SE	CP	RE
n=80	Integrating 10 outcomes	β_0	0.1	12.3	10.5	90	0.9	0.1	11.7	10.9	92	1.0
		β_1	0.3	13.1	9.8	85	1.1	-0.3	13.5	11.4	91	1.0
		β_2	-0.2	13.9	10.5	85	1.2	0.0	14.4	12.2	90	1.1
		β_3	-0.3	11.7	8.3	84	1.7	-0.5	11.5	9.4	89	1.7
		β_4	0.4	9.7	7.5	86	1.9	0.2	9.9	8.4	90	1.8
	Integrating 50 outcomes	β_0	-0.3	17.9	10.8	82	0.4	0.0	12.0	11.0	93	0.9
		β_1	0.4	25.5	8.7	70	0.3	-0.6	11.6	8.9	86	1.5
		β_2	1.4	43.1	9.5	68	0.1	0.3	11.8	9.7	89	1.7
		β_3	-1.7	49.1	9.6	68	0.1	-0.2	12.3	9.7	89	1.6
		β_4	-0.7	20.7	9.2	69	0.4	-0.5	11.2	9.0	88	1.4
n=150	Integrating 10 outcomes	β_0	-0.2	8.5	7.9	93	1.0	-0.2	8.4	8.0	94	1.0
		β_1	0.0	8.4	7.3	91	1.3	-0.4	9.2	8.6	93	1.1
		β_2	-0.5	9.4	7.8	90	1.5	-0.4	10.5	9.1	90	1.2
		β_3	0.2	7.2	6.1	90	2.4	0.3	7.7	6.7	92	2.1
		β_4	-0.1	6.5	5.4	90	2.2	0.0	6.7	5.9	92	2.0
	Integrating 50 outcomes	β_0	-0.2	9.0	7.6	90	0.9	-0.2	8.5	8.1	94	1.0
		β_1	0.3	6.6	5.2	87	2.0	0.0	7.1	6.4	92	1.8
		β_2	0.3	7.6	5.5	84	2.1	0.3	7.8	6.8	92	2.0
		β_3	0.1	7.1	5.5	87	2.2	0.1	7.3	6.7	93	2.0
		β_4	-0.1	6.9	5.2	86	2.0	0.0	7.2	6.4	92	1.8

Abbreviations: MCSD, Monte Carlo standard deviation; SE, estimated standard error; RE, relative efficiency over the naive estimator; CP, 95% coverage probability.

4 More details on data application

In this section we present more details of the data application in the manuscript.

4.1 Biomarkers list

In the data application, 25 biomarkers were taken into consideration. In particular, we considered 22 blood biomarkers: Alanine aminotransferase, SHBG, Triglycerides, HDL cholesterol, C-reactive protein, Glycated haemoglobin, Gamma glutamyltransferase, Testosterone, Urate, Calcium, Vitamin D, IGF-1, Cholesterol, Total bilirubin Blood, Glucose, Alkaline phosphatase, Total protein, Creatinine, Cystatin C, Urea, Phosphate, and Albumin. Besides, we considered 3 urine biomarkers: Sodium in urine, Creatinine (enzymatic) in urine, and Potassium in urine.

4.2 Profile of the matched cohort

The profiles of the matched cohort for the analysis of LBRS and PDFF are summarized in Tables S8 and S9, respectively. Examining the tables, we could infer that the population characteristics were close.

Table S8: Profile of the matched cohort of the LBRS analysis in the data application.

Covariates		Heavy smokers (N=1893)	Others (N=1893)	Overall (N=3786)
Age	Below 65	1654 (87.4%)	1654 (87.4%)	3308 (87.4%)
	No less than 65	239 (12.6%)	239 (12.6%)	478 (12.6%)
Sex	Female	749 (39.6%)	767 (40.5%)	1516 (40.0%)
	Male	1144 (60.4%)	1126 (59.5%)	2270 (60.0%)
Education	College or university degree	907 (47.9%)	871 (46.0%)	1778 (47.0%)
	Others	986 (52.1%)	1022 (54.0%)	2008 (53.0%)
Income	Below £31,000 annually	597 (31.5%)	590 (31.2%)	1187 (31.4%)
	No less than £31,000 annually	1296 (68.5%)	1303 (68.8%)	2599 (68.6%)
BMI	Mean (SD)	27.2 (4.16)	27.2 (4.46)	27.2 (4.31)
	Median [Min, Max]	26.7 [16.7, 47.8]	26.5 [18.0, 49.0]	26.6 [16.7, 49.0]
Activity	Mean (SD)	10.4 (4.70)	10.2 (4.63)	10.3 (4.66)
	Median [Min, Max]	10.0 [0, 21.0]	10.0 [0, 21.0]	10.0 [0, 21.0]
SBP	Mean (SD)	136 (18.6)	136 (18.1)	136 (18.3)
	Median [Min, Max]	135 [90.5, 226]	134 [95.0, 237]	134 [90.5, 237]
DBP	Mean (SD)	81.6 (9.90)	81.8 (9.92)	81.7 (9.91)
	Median [Min, Max]	81.0 [50.0, 123]	81.5 [54.0, 133]	81.5 [50.0, 133]

Table S9: Profile of the matched cohort of the liver PDFF validation analysis in the data application.

Covariates		Heavy smokers (N=1893)	Others (N=1893)	Overall (N=3786)
Age	Below 65	458 (85.0%)	458 (85.0%)	916 (85.0%)
	No less than 65	81 (15.0%)	81 (15.0%)	162 (15.0%)
Sex	Female	233 (43.2%)	224 (41.6%)	457 (42.4%)
	Male	306 (56.8%)	315 (58.4%)	621 (57.6%)
Education	College or university degree	215 (39.9%)	208 (38.6%)	423 (39.2%)
	Others	324 (60.1%)	331 (61.4%)	655 (60.8%)
Income	Below £31,000 annually	210 (39.0%)	217 (40.3%)	427 (39.6%)
	No less than £31,000 annually	329 (61.0%)	322 (59.7%)	651 (60.4%)
BMI	Mean (SD)	27.6 (4.37)	27.4 (4.50)	27.5 (4.43)
	Median [Min, Max]	27.1 [17.6, 48.3]	26.8 [18.0, 52.0]	27.0 [17.6, 52.0]
Activity	Mean (SD)	10.2 (4.93)	10.3 (4.79)	10.3 (4.86)
	Median [Min, Max]	10.0 [0, 21.0]	10.0 [0, 21.0]	10.0 [0, 21.0]
SBP	Mean (SD)	137 (18.0)	136 (18.8)	137 (18.4)
	Median [Min, Max]	136 [96.0, 196]	135 [87.0, 221]	136 [87.0, 221]
DBP	Mean (SD)	82.3 (9.33)	82.1 (10.4)	82.2 (9.89)
	Median [Min, Max]	82.0 [57.0, 109]	82.0 [51.5, 119]	82.0 [51.5, 119]

4.3 Additional validation analysis

We conducted additional validation analysis where the liver PDFF values were predicted only by the blood and urine biomarkers, and the remaining procedures were the same. The sample size of the training data with complete measurements of biomarkers and liver PDFF is $3,109$. The results are summarized in Table S10. Compared with the results in the manuscript, we could infer that most results were consistent. Notably, the efficiency gain was even larger than the primary analysis.

4.4 Sensitivity analysis on training the prediction model

In the main manuscript, we considered 5-fold cross validation to tune the prediction model for liver PDFF in data application. In this section, we considered 10 and 15-fold cross validation to assess the robustness of our findings. The results (Tables S11 and S12) were

Table S10: Results of the naive OLS and PEPSI models for estimating the effect of smoking on liver PDFF with prediction model trained by secondary outcomes.

		OLS					PEPSI					
		EST	SE	LL	UL	P	EST	SE	LL	UL	P	RE
Regression parameters	Intercept	5.598	0.075	5.451	5.746	0.000	5.582	0.055	5.475	5.689	0.000	1.906
	Smoking	0.064	0.106	-0.144	0.272	0.545	0.066	0.046	-0.024	0.156	0.151	5.329
	Smoking:Age	0.101	0.276	-0.441	0.642	0.716	0.368	0.109	0.154	0.582	0.001	6.383
	Age	-0.195	0.195	-0.577	0.187	0.317	-0.263	0.089	-0.437	-0.088	0.003	4.792
Effect of smoking	Age below 65	0.064	0.106	-0.144	0.272	0.545	0.066	0.046	-0.024	0.156	0.151	5.329
	Age no less than 65	0.165	0.255	-0.335	0.664	0.518	0.434	0.101	0.235	0.632	0.000	6.333
	Overall	0.077	0.098	-0.115	0.269	0.433	0.112	0.043	0.029	0.196	0.008	5.281

Abbreviations: EST, parameter estimates; SE, estimated standard errors; LL & UL, 95% lower and upper confidence limits of estimates; P, p-values; RE, relative efficiency, ratios of estimated variances between naive OLS and PEPSI model estimates.

consistent with our findings presented in the main manuscript (Table 3).

Table S11: Results of the naive OLS and PEPSI models for estimating the effect of smoking on liver PDFF with prediction model trained by 10-fold cross validation.

		OLS					PEPSI					
		EST	SE	LL	UL	P	EST	SE	LL	UL	P	RE
Regression parameters	Intercept	5.433	0.075	5.285	5.580	0.000	5.404	0.056	5.294	5.514	0.000	1.802
	Smoking	0.054	0.104	-0.151	0.259	0.603	0.068	0.052	-0.034	0.170	0.193	4.043
	Smoking:Age	0.103	0.261	-0.409	0.615	0.693	0.280	0.116	0.053	0.507	0.016	5.099
	Age	-0.222	0.182	-0.580	0.135	0.223	-0.231	0.090	-0.408	-0.054	0.010	4.086
Effect of smoking	Age below 65	0.055	0.106	-0.154	0.264	0.605	0.063	0.052	-0.040	0.166	0.229	4.138
	Age no less than 65	0.157	0.239	-0.312	0.627	0.511	0.347	0.105	0.141	0.554	0.001	5.180
	Overall	0.067	0.096	-0.121	0.256	0.484	0.103	0.048	0.009	0.197	0.031	4.056

Abbreviations: EST, parameter estimates; SE, estimated standard errors; LL & UL, 95% lower and upper confidence limits of estimates; P, p-values; RE, relative efficiency, ratios of estimated variances between naive OLS and PEPSI model estimates.

4.5 Sensitivity analysis on accounting for more confounders

In the main manuscript, we considered a variety of baseline covariates to predict liver PDFF and to do matching to remove confounding effects. However, the effect of smoking on PDFF is complex, and many confounders need to be accounted for. Therefore, we conducted a sensitivity analysis where we included more baseline covariates. Specifically, we considered the diet habit (average Z-scores of monthly consumptions of raw vegetables, cooked vegetables, fresh fruits, and dried fruits) and the previous drinking habit (three

Table S12: Results of the naive OLS and PEPSI models for estimating the effect of smoking on liver PDFF with prediction model trained by 15-fold cross validation.

		OLS					PEPSI					
		EST	SE	LL	UL	P	EST	SE	LL	UL	P	RE
Regression parameters	Intercept	5.479	0.075	5.331	5.626	0.000	5.451	0.056	5.341	5.561	0.000	1.809
	Smoking	0.049	0.104	-0.155	0.253	0.639	0.058	0.051	-0.043	0.159	0.261	4.097
	Smoking:Age	0.102	0.260	-0.407	0.612	0.694	0.270	0.116	0.042	0.498	0.020	5.015
	Age	-0.227	0.182	-0.584	0.130	0.212	-0.224	0.091	-0.402	-0.045	0.014	3.992
Effect of smoking	Age below 65	0.049	0.104	-0.155	0.253	0.639	0.058	0.051	-0.043	0.159	0.261	4.097
	Age no less than 65	0.151	0.238	-0.316	0.618	0.526	0.328	0.106	0.120	0.536	0.002	5.039
	Overall	0.062	0.096	-0.126	0.250	0.519	0.092	0.047	-0.001	0.185	0.053	4.086

Abbreviations: EST, parameter estimates; SE, estimated standard errors; LL & UL, 95% lower and upper confidence limits of estimates; P, p-values; RE, relative efficiency, ratios of estimated variances between naive OLS and PEPSI model estimates.

times a week or more vs. others). Most of the results (Table S13) were consistent with our findings presented in the main manuscript (Table 3), indicating the robustness of our conclusions.

Table S13: Results of the naive OLS and PEPSI models for estimating the effect of smoking on liver PDFF adjusting for more confounders.

		OLS					PEPSI					
		EST	SE	LL	UL	P	EST	SE	LL	UL	P	RE
Regression parameters	Intercept	5.417	0.071	5.278	5.556	0.000	5.423	0.051	5.322	5.524	0.000	1.892
	Smoking	0.080	0.099	-0.113	0.274	0.415	0.012	0.046	-0.078	0.102	0.792	4.620
	Smoking:Age	0.137	0.249	-0.350	0.625	0.580	0.230	0.106	0.022	0.438	0.030	5.482
	Age	-0.270	0.175	-0.613	0.072	0.122	-0.265	0.081	-0.423	-0.106	0.001	4.659
Effect of smoking	Age below 65	0.080	0.099	-0.113	0.274	0.415	0.012	0.046	-0.078	0.102	0.792	4.620
	Age no less than 65	0.218	0.228	-0.229	0.665	0.339	0.242	0.096	0.053	0.430	0.012	5.621
	Overall	0.098	0.091	-0.080	0.276	0.282	0.041	0.042	-0.041	0.124	0.327	4.676

Abbreviations: EST, parameter estimates; SE, estimated standard errors; LL & UL, 95% lower and upper confidence limits of estimates; P, p-values; RE, relative efficiency, ratios of estimated variances between naive OLS and PEPSI model estimates.

4.6 External validation analysis

In the main manuscript, we considered the UKB data to investigate the effect of heavy smoking on liver PDFF. In this section, we performed an external validation of our findings using the Alzheimer's Disease Neuroimaging Initiative (ADNI) dataset [Mueller et al., 2005]. Compared with the UKB data, the ADNI data had smaller sample size and less

comprehensive clinical factors and biomarkers measurements. Therefore, we re-conducted the training and testing using these two datasets.

Specifically, based on the variables available in both datasets, we considered age, sex, education, BMI, systolic blood pressure, diastolic blood pressure, smoking, and 6 blood biomarkers: Cholesterol, HDL cholesterol, Triglycerides, Albumin, and Alkaline phosphatase. The missing rates for these variables were no more than 30%. Therefore, we imputed the missing values using multiple imputation by chained equations [Van Buuren and Groothuis-Oudshoorn, 2011], where we imputed 25 times and took average (rounded up for binary variables). The remaining process followed the same steps as in the main manuscript. We first trained a model to predict liver PDFF using the UKB data and then applied this model to the ADNI data to predict liver PDFF values. Within the ADNI data, heavy smokers and non-smokers were matched based on age, sex, education, BMI, systolic blood pressure, and diastolic blood pressure. After matching, the covariates were balanced across groups and there were 474 people in each group, with 93% elders. Finally we performed regression analyses to estimate the effect of smoking.

Table S14: Results of the naive OLS and PEPSI models for estimating the effect of smoking on liver PDFF using the ADNI dataset as an external validation data.

		OLS					PEPSI					
		EST	SE	LL	UL	P	EST	SE	LL	UL	P	RE
Regression parameters	Intercept	4.539	0.394	3.766	5.311	0.000	4.733	0.261	4.221	5.246	0.000	2.275
	Smoking	0.439	0.593	-0.722	1.601	0.459	0.130	0.372	-0.598	0.859	0.726	2.541
	Smoking:Age	-0.545	0.608	-1.737	0.647	0.370	-0.084	0.384	-0.837	0.669	0.827	2.507
	Age	-0.266	0.407	-1.063	0.531	0.513	-0.545	0.268	-1.070	-0.020	0.042	2.305
Effect of smoking	Age below 65	0.439	0.593	-0.722	1.601	0.459	0.130	0.372	-0.598	0.859	0.726	2.541
	Age no less than 65	-0.106	0.136	-0.373	0.161	0.438	0.047	0.098	-0.145	0.238	0.633	1.948
	Overall	-0.069	0.133	-0.330	0.192	0.605	0.052	0.095	-0.133	0.238	0.580	1.987

Abbreviations: EST, parameter estimates; SE, estimated standard errors; LL & UL, 95% lower and upper confidence limits of estimates; P, p-values; RE, relative efficiency, ratios of estimated variances between naive OLS and PEPSI model estimates.

Most of the results (Table S14) were consistent with our findings presented in the main manuscript. Specifically, the RE of the PEPSI estimator was 2 to 3 times compared

with the standard estimator, further identifying elder people had significantly lower liver PDFF. It indicated the generalizability of our framework. However, some estimates, such as the interaction of smoking and age, and the effects of smoking for elders and overall population, were not significant, which could be due to the relatively small sample size and the uncertainty in multiple imputation.

References

T. W. Anderson. Asymptotic theory for principal component analysis. *The Annals of Mathematical Statistics*, 34(1):122–148, 1963.

P. J. Bickel, C. A. Klaassen, P. J. Bickel, Y. Ritov, J. Klaassen, J. A. Wellner, and Y. Ritov. *Efficient and adaptive estimation for semiparametric models*, volume 4. Springer, 1993.

J. Fan and R. Li. Variable selection via nonconcave penalized likelihood and its oracle properties. *Journal of the American statistical Association*, 96(456):1348–1360, 2001.

C. Leng and C. Y. Tang. Penalized empirical likelihood and growing dimensional general estimating equations. *Biometrika*, 99(3):703–716, 2012.

S. G. Mueller, M. W. Weiner, L. J. Thal, R. C. Petersen, C. Jack, W. Jagust, J. Q. Trojanowski, A. W. Toga, and L. Beckett. The alzheimer's disease neuroimaging initiative. *Neuroimaging Clinics*, 15(4):869–877, 2005.

W. K. Newey and D. McFadden. Large sample estimation and hypothesis testing. *Handbook of econometrics*, 4:2111–2245, 1994.

A. B. Owen. *Empirical likelihood*. CRC press, 2001.

J. Qin and J. Lawless. Empirical likelihood and general estimating equations. *the Annals of Statistics*, 22(1):300–325, 1994.

J. Qin and J. Lawless. Estimating equations, empirical likelihood and constraints on parameters. *Canadian Journal of Statistics*, 23(2):145–159, 1995.

D. Shen, H. Shen, and J. S. Marron. A general framework for consistency of principal component analysis. *Journal of Machine Learning Research*, 17(150):1–34, 2016.

C. Y. Tang and C. Leng. Penalized high-dimensional empirical likelihood. *Biometrika*, 97 (4):905–920, 2010.

A. A. Tsiatis. *Semiparametric theory and missing data*. Springer, 2006.

S. Van Buuren and K. Groothuis-Oudshoorn. mice: Multivariate imputation by chained equations in r. *Journal of statistical software*, 45:1–67, 2011.

A. W. Van der Vaart. *Asymptotic statistics*, volume 3. Cambridge university press, 2000.
